# Global trends and hotspots in research on osteoporosis rehabilitation: A bibliometric study and visualization analysis

**DOI:** 10.3389/fpubh.2022.1022035

**Published:** 2022-11-30

**Authors:** Liqiong Wang, Jiaojiao Jiang, Yi Li, Jinming Huang, Renjie Wang, Yuxiang Liang, Chengqi He, Shaxin Liu

**Affiliations:** ^1^Rehabilitation Medicine Center, West China Hospital, Sichuan University, Chengdu, China; ^2^Rehabilitation Key Laboratory of Sichuan Province, West China Hospital, Sichuan University, Chengdu, China

**Keywords:** osteoporosis, rehabilitation, physical activity, bibliometric analysis, CiteSpace, VOSviewer

## Abstract

**Background:**

The field of rehabilitation medicine plays an essential role in the comprehensive management of osteoporosis and its consequences. The benefits of therapeutic exercise are increasingly being recognized in this area, which receives an increasing number of publications. this study was designed to comprehensively identify collaborative networks, parse and track research trends, spotlight present hotspots, and accurately predict frontiers and focus on the health topics related to osteoporosis rehabilitation.

**Methods:**

This research adopted computer retrieval of osteoporosis rehabilitation-related research published in the Web of Science Core Collection (WoSCC) from inception to June 14, 2022. The bibliometric visualization and comparative analysis involving countries, institutions, journals, authors, references, and keywords were performed using the CiteSpace and VOSviewer software.

**Results:**

A total of 3,268 articles were included, and the number of articles published each year has demonstrated a steady increase. The United States and the University of Melbourne were the highest productive country and institution, with 1,325 and 87 articles, respectively. The journal of osteoporosis international has published the greatest number of articles, with 221 publications, and the journal of bone and mineral research ranked first in the co-citation counts (cited by 11,792 times). The most productive and highly-cited authors were Heinonen A and Cummings S, with 35 publications and 680 citations.

**Conclusions:**

At present, “physical activity,” “weight bearing exercise,” “muscle strength,” “whole body vibration,” “postmenopausal women,” “older women,” children, men are the noteworthy research hot topics. Future research that focus on the major modes and parameters of physical activity/exercise for osteoporosis (including whole body vibration, weight bearing exercises, resistance training), targeted multicomponent training regimens, rehabilitation therapy for postmenopausal women, older women, children and men, osteoporosis related-sarcopenia and fractures, and mesenchymal stem cells are becoming frontiers and focus on the health topics related to osteoporosis rehabilitation in the upcoming years, which are worthy of further exploration.

## Introduction

Osteoporosis is a multifactorial disorder of bone metabolism associated with low bone mineral density and impaired microarchitecture that results in increased skeletal fragility and susceptibility to bone fracture (1). Consequently, patients who suffer from osteoporosis and fragility fractures frequently experience disability and therefore have decreased independence in daily life, and a low health-related quality of life (2–4). According to the National Osteoporosis Foundation, 10.2 million Americans suffer from osteoporosis, and an additional 43.4 million have decreased bone mass. It is predicted that by 2030, there will be will increase to 71 million low bone density and osteoporosis among adults (5), And more than nine million fractures are caused by osteoporosis worldwide every year one in five men and one in two women who are 50 years old will experience an osteoporotic fracture in their lifetime. Among women, the 10-year risk of developing fracture rises from 9.8 at 50 years to 21.7% at the age of 80 (6). Osteoporotic fractures often required hospitalization, especially hip fractures, which commonly account for 50% of osteoporotic fracture-related hospitalizations (7). Once in hospital, these patients have a high risk of complications including thrombosis (27%), urinary tract infections (12–61%), and pneumonia (7 %) (7), which increase the mortality and morbidity of patients with osteoporotic fractures. Therefore, osteoporosis and osteoporotic fractures pose a heavy public health burden on society. The research (7) showed that the average cost of a patient with a fracture in a hospital is close to $13,000 for all sorts of fractures.

The prevention and treatment of osteoporosis should incorporate both non-pharmacological and pharmacological approaches in order to minimize fracture risk (8). It is noteworthy that rehabilitation medicine takes a primary role in the comprehensive management of osteoporosis and its consequences, taking into account the benefits of therapeutic exercise for fall prevention as well as the functional recovery after fragility fractures, and it also can affect bone metabolism and thus enhance bone strength. Consequently, these fields receive an increasing number of publications. Exercise training interventions are recommended for osteoporosis management in many guidelines. Many clinical guidelines strongly recommend weight-bearing, muscle-strengthening exercises for maintaining bone health, preventing bone loss and falls, reducing discomfort (9–13), improving quality of life and according to Exercise and Sports Science Australia (ESSA) (14) evidence, muscle strength, balance, and mobility training also helps to minimize the risk of falling. Additionally, studies have evaluated the guidelines for osteoporosis using the AGREE II tool. The results showed that patients with osteoporosis and fragility fractures may benefit from moderate intensity therapeutic exercise, according to some guidelines, and a majority of the guidelines were high quality (15). In addition, there have been a large number of randomized controlled clinical trials and review that have demonstrated resistance training (RT) and impact training, whole-body vibration training, balance exercise programme can improve bone mineral density (BMD), physical function, muscle strength, balance and reduce fear of falling and pain (8, 16–23). However the majority of the studies discussed above have focused on specific and limited aspects. Research on osteoporosis rehabilitation and prevention, and the general overview of this field is lacking.

Bibliometric visualization analysis is a quantitative analysis integrating mathematics and statistical methodologies, which can assist researchers in understanding the characteristics of the field's development over time (24). It is possible to perform an in-depth assessment of research trends and the focus of a specific topic employing comprehensive indexes such as journals, authors, countries, and institutions (24, 25). Authors with a high number of total citations are recognized for their scientific achievements by their peers (26). In addition, bibliometric evaluation results can also provide suggestions for further research and decision-making. CiteSpace visual analysis software produces co-citation networks based on reference citations to expose the structure of a research field, enabling visual knowledge discovery in bibliographic databases. VOS viewer is another scientometric application for producing and viewing network maps. Those knowledge maps can illustrate the output of authors and institutions, cooperation linkages, geographic dispersion, the most cited and critical documents, the emergence of research topics, highlighting disciplinary development and research tendencies within a particular area. In recent years, CiteSpace and VOS viewer have been used in a variety of fields, including medical treatment, machine learning, cities or communities research, agriculture and environment management (27–31). There are also currently studies evaluating bisphosphates for osteoporosis, research trends on male osteoporosis, miRNAs in osteoporosis-related research (32–34). However, there is no targeted bibliometric analysis of worldwide scientific studies on rehabilitation therapies for osteoporosis. Therefore, the aim of this study is to provide a summary of the global research base, as well as hotspots and frontiers of rehabilitation treatment for osteoporosis through bibliometric analysis.

## Materials and methods

### Data source and search strategy

**Figure 1:**
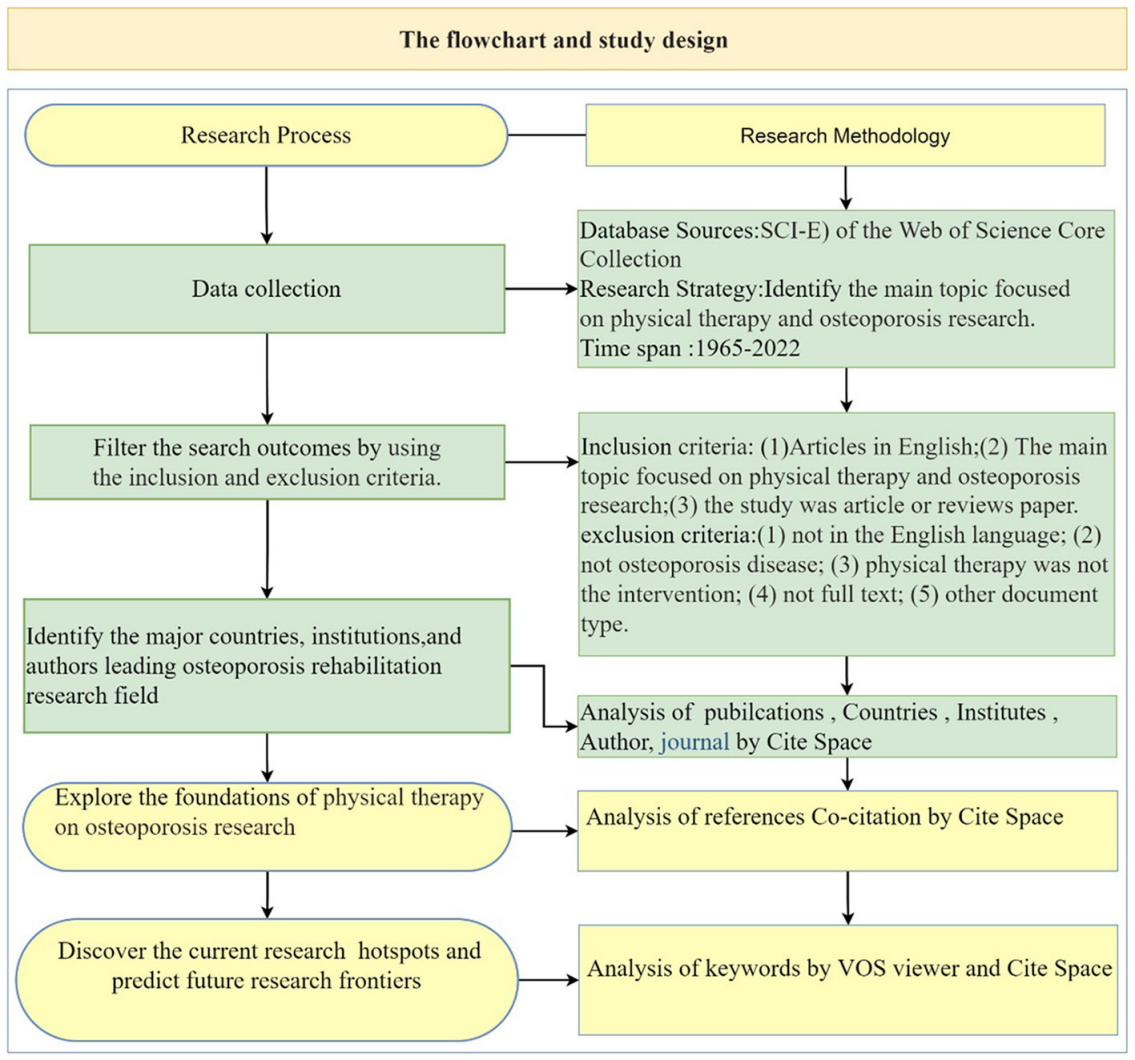
The summary of the flowchart and study design.

All published literature was obtained online from the Science Citation Index-Expanded (SCI-E) of the Web of Science Core Collection (WoSCC) on June 14, 2022.The following retrieval strategy were conducted for search publications (Supplementary Table S1), which the main topic focused on physical therapy and osteoporosis research. The range of publication dates was selected from inception to June 14, 2022. According to the previous bibliometric research (35, 36). The “articles or reviews” were chosen for analysis, and the language was limited to “English,” non-English articles and other document type, such as conference abstracts, letters, reviews, news, were excluded. An overview of the comprehensive search approach and inclusion criteria used in this research was showed in Figure 1.

### Literature screening and data extraction

Screening was conducted in two stages, namely, preliminary screening was conducted according to the title and abstract, and then full-text screening was carried out using the inclusion and exclusion criteria. Studies were included should meet all of the following criteria: (1) Articles in English; (2) The main topic focused on physical therapy and osteoporosis research; (3) the study was article or reviews paper. Accordance with the following exclusion criteria, records were excluded: (1) not in the English language; (2) not osteoporosis disease; (3) physical therapy was not used for the intervention programs; (4) not full text; (5) other document type: conference abstracts, letters, news, editorial materials, proceeding papers, short reports, case studies.

### Data analysis tool and statistical methods

VOS viewer 1.6.18 (Leiden University, Van Eck NJ) and CiteSpace 6.1.R2 (Drexel University, Philadelphia, PA, United States) were used for the statistical analyses of extracted literature. Visualized networks by VOS viewer based on bibliographic data were presented, including the full counting bibliographic coupling analysis of journals, co-occurrence of all keywords, co-citation analysis of cited journals in the references. The following parameters are chosen: counting method (full counting), Type of Analysis (co-occurrence), Unit of Analysis (All Keywords), Minimum number of occurrences of a keyword (5), Number of keywords to be selected (1000).

CiteSpace is an important bibliometric analysis software, we investigated the primary areas of osteoporosis rehabilitation therapy, as well as research hotspots and frontiers by it. The parameters are set as follows; time slicing (1965–2022), years per slice (1), term source (all chosen), node type (one at a time), the threshold (top *N* = 50), pruning (Pathfinder, pruning sliced networks), and visualization (cluster view-static, show merged network). In order to create the co-citation knowledge graph, authors, institutions, countries, cited authors were chosen for node type co-occurrence analysis. the size of the node denotes the number or frequency of documents. The relationships between nodes are represented by their connections, such as coexistence, co-occurrence, or co-citation (37, 38). The purple outer ring of the circle represents centrality, the wider the circle, the higher is the centrality. In addition, reference citation clusters analysis and citation bursts were performed, and the top 10 paper with the highest cited frequency and strongest citation bursts were also summarized. Reference co-citation clusters were generated using the log-likelihood ratio (LLR) strategy, with the selection type adopted “keyword option.” The modularity value (Q-value) and the weighted mean silhouette value (S-value) were used as clustering criteria. As for keyword analysis, the top 30 keywords are summarized, and the top 30 keywords with the strongest citation bursts also were diagrammed.

## Results

### The number and growth trend of annual publications

A total of 3,268 articles were included from 1965 to 2022, the number of publications was summarized in Supplementary Table S2. The numbers of papers published each year and the development tendency on physical therapy of osteoporosis health promotion field is shown in Supplementary Figure S1. Overall, the number of published papers increased steadily during three stages. In the first stage (1965–1990), publications remained at a low level of no more than 10 per year before 1990. After that, the number of published papers climbed from 35 in 1991 to 102 in 2006, and broke through 100 for the first time in 2006 in the second stage (1991–2006). The period from 2007 to 2022 was the third stage, the annual publication rapidly increased and the average number of published articles was (136.62 ± 32.25), accounting for 66.89% of the total publications. Since only 7 months were included for 2022, the numbers dropped slightly.

### Distribution of countries and institutes

**Figure 2:**
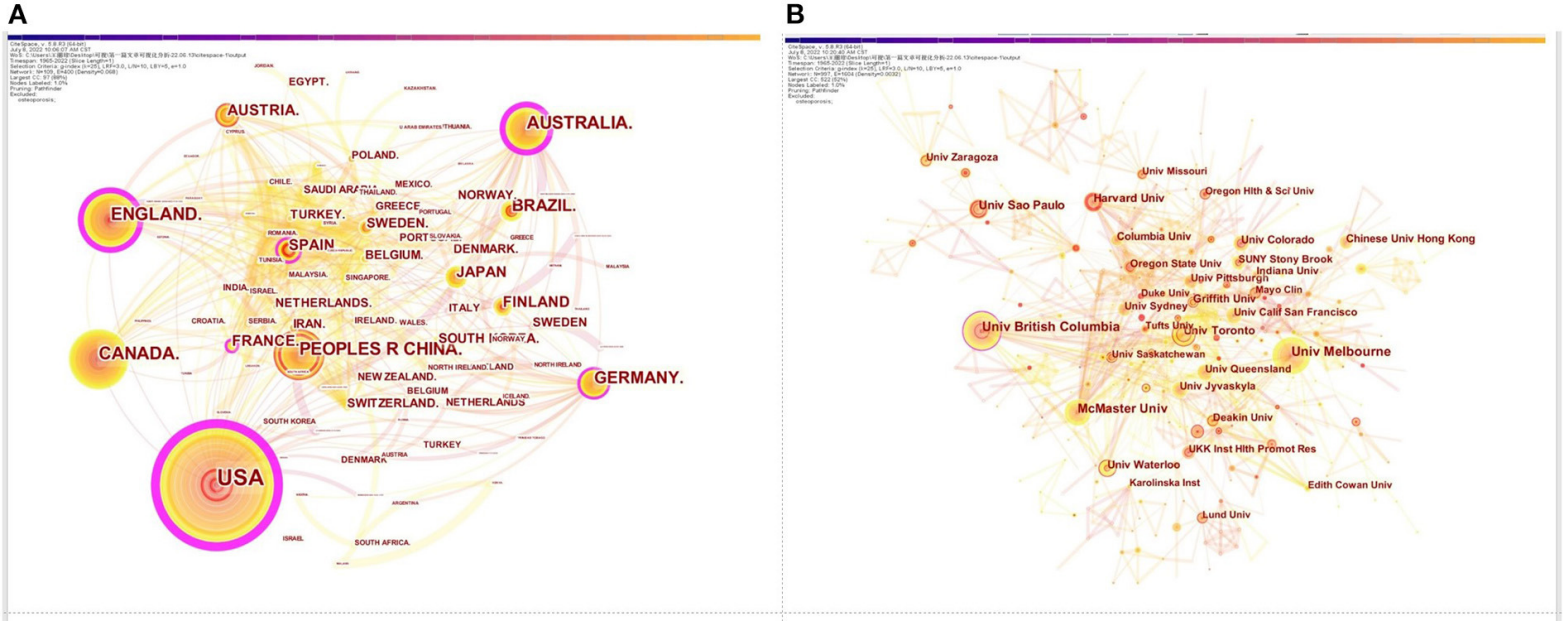
Cooperation network diagram of countries **(A)**; Cooperation network diagram of institutions **(B)**.

The details of the top 10 countries and institutions in terms of the numbers of publications are showed in Table 1. The US had the most publications (1,325), followed by China (390), Australia (303), the UK (293), Canada (286), and Germany (179 publications). Articles published in the above six countries accounted for 84.94% of the total articles. The collaborations among various countries were showed in Figure 2A. United States had the highest centrality (0.52), which was followed by United Kingdom (0.25) and Australia (0.21). According to the relevant definition of centrality, these countries had close cooperation with other countries and represented tremendous academic influence. When publications and centrality analysis were combined, United States, United Kingdom and Australia were in the dominant positions. The top 10 institutions by the amounts of published papers were listed in Table 1. University of Melbourne (87 publications) was the leading institution, followed by University of British Columbia (86 publications), McMaster University (70 publications), University of São Paulo (51 publications), Harvard University (49 publications). The network cooperation map among various institutions were depicted in Figure 2B. In terms of centrality, University of British Columbia showed maximum centrality (0.11), followed by Harvard University (0.09), McMaster University (0.08). Based on the analysis of the number and centrality of publications, University of British Columbia, Harvard University and McMaster University showed close cooperative relationship and exhibited strong academic influence.

**Table 1:** Ranking of top 10 countries and institutions involved in the osteoporosis rehabilitation field.

**Rank**	**Country**	**Publications**	**Centrality**	**Institution**	**Publications**	**Centrality**
1	United States	1,325	0.52	University of Melbourne	87	0.05
2	China	390	0.01	University of British Columbia	86	0.11
3	Australia	303	0.21	McMaster University	70	0.08
4	United Kingdom	293	0.25	University of São Paulo	51	0.04
5	Canada	286	0.04	Harvard University	49	0.09
6	Germany	179	0.19	University of Toronto	48	0.04
7	Brazil	152	0	University of Waterloo	40	0.01
8	Japan	145	0.04	University of Colorado	38	0.01
9	Austria	141	0.07	Griffith University	37	0.01
10	Spain	131	0.15	Columbia University	36	0.03

### Distribution of authors and co-cited authors

**Figure 3:**
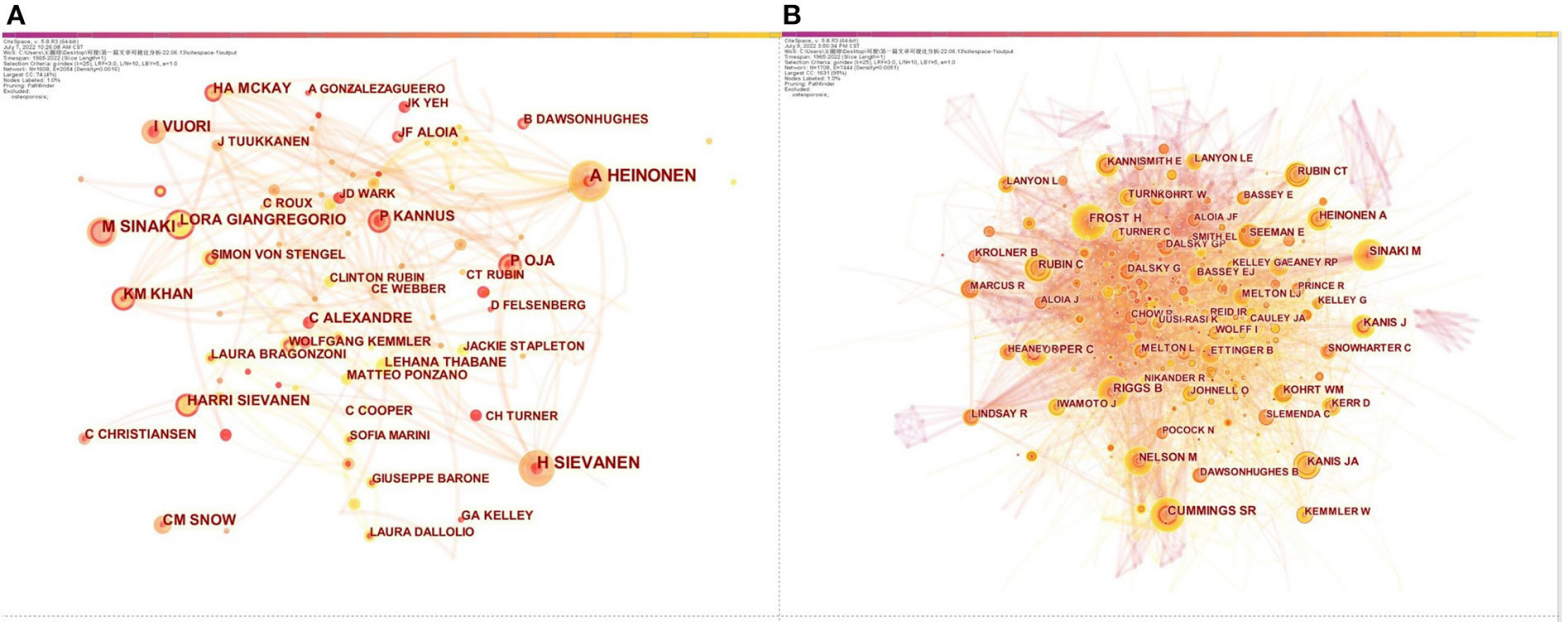
Network diagram of influential authors **(A)**; Network diagram of influential co-cited authors **(B)**.

The top 10 authors, co-cited authors on physical therapy of osteoporosis research were listed in Table 2. Among the top 10 authors, Heinonen A had the most publications (35 publications), followed by Sievanen H (31 publications), and Sinaki M (25 publications). In terms of co-cited authors, Cummings S had the most cited times (680 cited times), followed by Sinaki M (495 cited times) and Rubin C (472 cited times). These authors were actively involved in the physical therapy for osteoporosis research field. Cooperation between authors and co-cited authors is analyzed in Figures 3A,B. Connections showed cooperation relationships among nodes, the bigger the circle, the more it appears, and lines represents the connections between authors, the thicker the lines, the closer the connections. The network diagram of authors in Figure 3A is composed of 1,608 nodes and 2,054 links the connections between co-cited authors (nodes = 558, links = 3,288) was illustrated in Figure 3B. The maps of the authors and co-cited provided significant information on influential research collaborators, enabling close collaboration among researchers.

**Table 2:** Ranking of top 10 authors, co-cited authors in the physical therapy for osteoporosis research domain.

**No**.	**Author**	**N**	**Cited author**	**Frequency**
1	Heinonen A	35	Cummings S	680
2	Sievanen H	31	Sinaki M	495
3	Sinaki M	25	Rubin C	472
4	Vuori I	21	[Anonymous]	452
5	Khan KM	20	KANIS JA	429
6	Giangregorio L	19	Frost HM	391
7	Snow CM	18	Heinonen A	367
8	Oja P	17	Cooper C	357
9	Mckay HA	16	Kanis J	342
10	Kannus P	16	RUBIN CT	338

### Distribution of journals and co-cited journals

**Figure 4:**
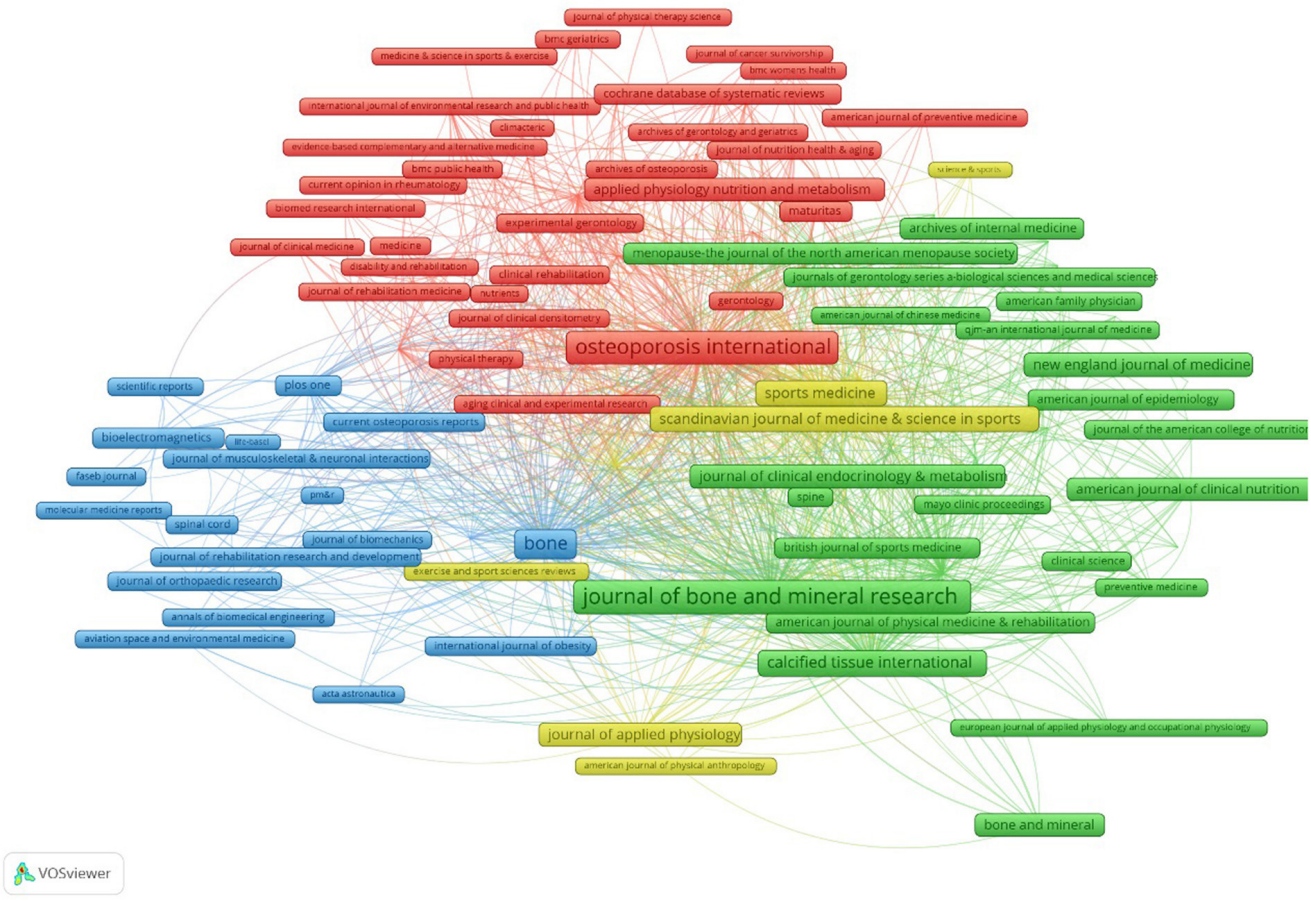
Bibliographic coupling analysis highly cited journals, weighted by citations, visualized map.

**Figure 5:**
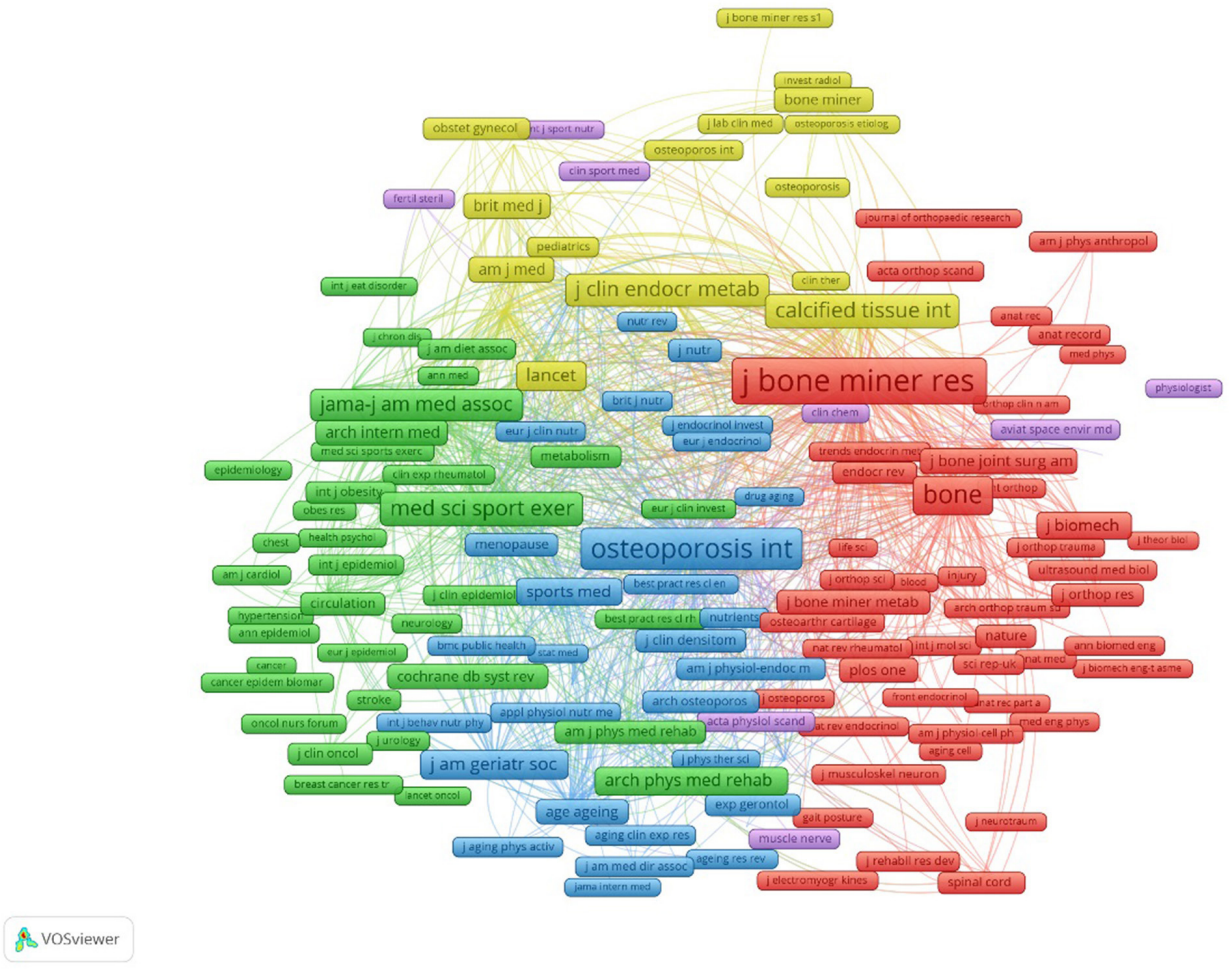
Co-citation analysis of most frequently cited journals in the reference lists, weighed by citations, visualized map.

The publications were concentrated published in 132 scholarly journals, according to the full counting bibliographic coupling analysis of journals (Figure 4). The top 10 journals that were most cited were listed in Table 3. Bone and Mineral Research had 11,222 citations and 123 publications, making it the most cited journal, followed by Osteoporosis International with 10,671 citations and 221 publications, the third is Bone with 6,621 citations and 133 articles. The most frequently cited in the references, according to the full counting co-citation analysis of cited journals, were Journal of Bone and Mineral Research (cited by 11,792 times). The second was Osteoporosis International (cited by 8,146 times). followed by Bone (cited by 6,396 times) (Figure 5). The top 10 cited journals in the references were showed in Table 3.

**Table 3:** List of the top 15 journals and cited journals in the references.

**NO**.	**Journal**	**Documents**	**Citations**	**Cited journal**	**Citations**
1	Journal of Bone and Mineral Research	123	11,222	Journal of Bone and Mineral Research	11,792
2	Osteoporosis International	221	10,671	Osteoporosis International	8,146
3	Bone	133	6,621	Bone	6,396
4	Medicine and Science in Sports and Exercise	63	5,543	Calcifiedtissue International	4,146
5	Calcified Tissue International	84	3,751	Medicine and Science in Sports and Exercise	3,682
6	Sports Medicine	31	3,209	Journal of Clinical Endocrinology & Metabolism	3,214
7	Scandinavian Journal of Medicine & Science in Sports	26	2,741	New England Journal of Medicine	3,177
8	New England Journal of Medicine	5	2,231	Jama-Journal of the American Medical Association	2,703
9	Journal of Clinical Endocrinology & Metabolism	22	2,063	Applied Physiology	2,614
10	Journal of Applied Physiology	35	1,973	American Journal of Clinical Nutrition	2,132
11	Archives of Physical Medicine and Rehabilitation	31	1,776	Journal of the American Geriatrics Society	1,920
12	American Journal of Clinical Nutrition	19	1,762	Lancet	1,809
13	Annals of Internal Medicine	6	1,572	Archives of Internal Medicine	1,679
14	Bone and Mineral	6	1,451	Archives of Physical Medicine and Rehabilitation	1,576
15	Applied Physiology Nutrition and Metabolism	13	1,420	Journals of Gerontology. Series A,Biological Sciences & Medical Sciences	1,307

### Analysis of reference co-citation

**Figure 6:**
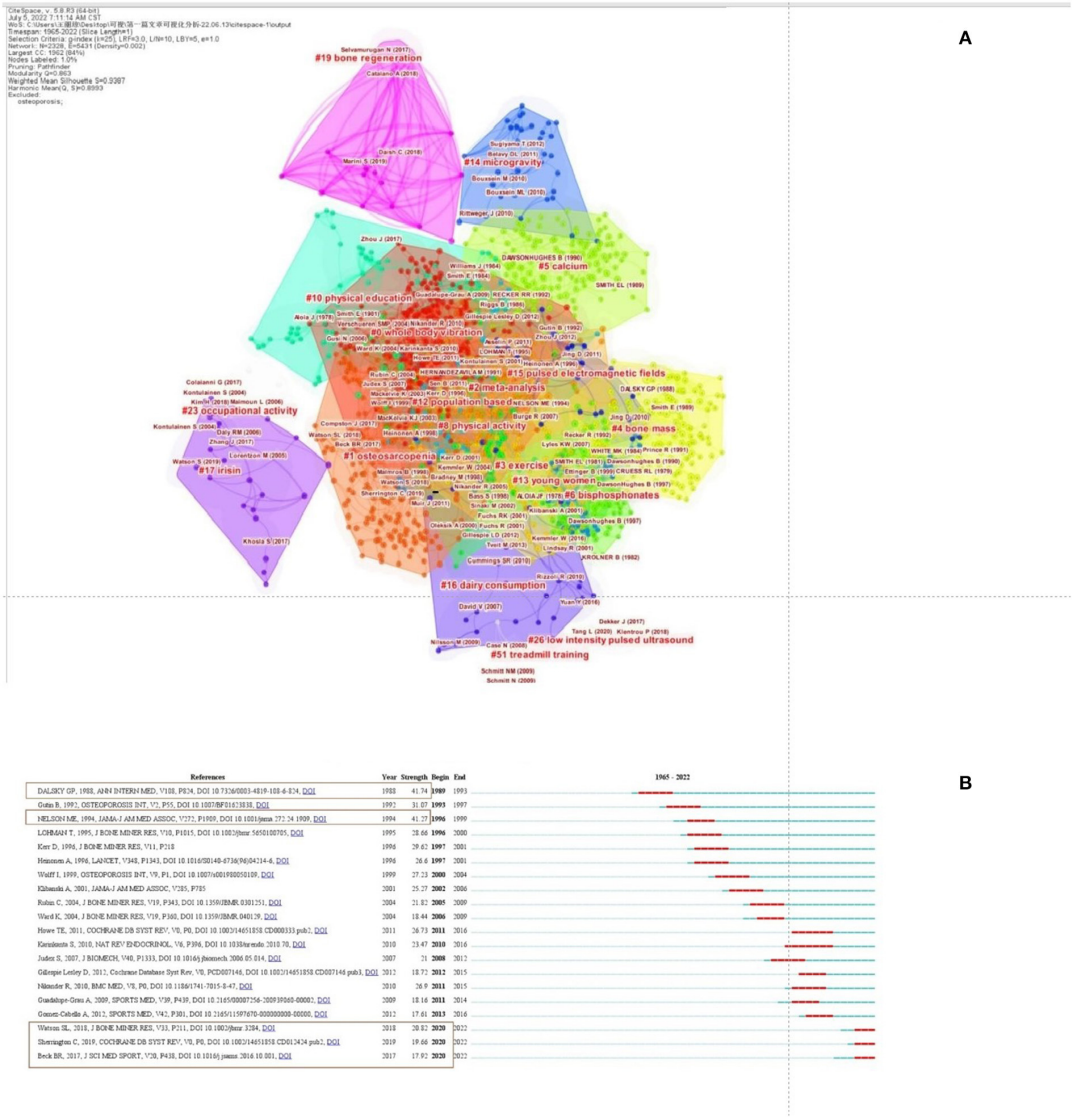
Co-citation clustering of osteoporosis rehabilitation studies **(A)**. The top 20 references with the strongest citation bursts on osteoporosis rehabilitation research **(B)**.

Reference co-citation analysis was utilized to explore research priorities in academic fields. The co-citation analysis gained 4,106 recorded and the co-citation network is shown in Figure 6A, Table 4. Reference co-citation clusters were generated using the log-likelihood ratio (LLR) strategy, with the selection type adopted “keyword option.” The Q-value and S-value were used as clustering criteria. Estimation strategies with means Q > 0.5 and S > 0.7 suggest that the clustering is convincing. In Figure 6A, the modularity Q is 0.863 (>0.5), indicating that the clustering of the network is reasonable, and the S-value is 0.9387 (>0.5), indicating that the homogeneity of the clustering is acceptable. References with the highest cited frequency and strongest citation bursts are regarded as the research basics of frontiers in the future. The top 20 papers with the strongest citation bursts on osteoporosis rehabilitation research were showed in Figure 6B. The important 10 articles integrating the cited frequency and burst strength were discovered (Table 5).

**Table 4:** Co-citation clustering of osteoporosis rehabilitation studies that were published from 1965 to 2022.

**Cluster ID**	**Size**	**Silhouette**	**Silhouette mean (Year)**	**Label (LLR)**
0	271	0.872	2009	Whole body vibration
1	211	0.983	2017	Osteosarcopenia
2	178	0.929	1995	Meta-analysis
3	168	0.892	2003	Exercise
4	159	0.945	1989	Bone mass
5	153	0.907	1991	Calcium
6	125	0.923	1997	Bisphosphonates
8	81	0.957	1999	Physical activity
10	72	0.994	1981	Physical education
12	55	0.97	2002	Population based
13	51	0.991	1980	Young women
14	37	0.965	2011	Microgravity
15	35	0.996	2012	Pulsed electromagnetic fields
16	32	0.992	2011	Dairy consumption
17	24	0.99	2018	Irisin
19	19	0.998	2017	Bone regeneration
23	11	0.997	2005	Occupational activity
26	10	0.992	2017	Low intensity pulsed ultrasound
51	4	0.998	2008	Treadmill training

**Table 5:** The top 10 papers with the highest cited frequency and strongest citation bursts on osteoporosis rehabilitation research.

**No**.	**Author/Year**	**Cited frequency**	**Burst**	**Centrality**	**Document title**	**Source**
1	(39)	81	41.27	0.02	Effects of high-intensity strength training on multiple risk factors for osteoporotic fractures: a randomized controlled trial	JAMA-J AM Med Assoc
2	(40)	73	26.73	0.04	Exercise for preventing and treating osteoporosis in postmenopausal women.	Cochrane DB Syst Rev
3	(41)	69	41.74	0.09	Weight-bearing exercise training and lumbar bone mineral content in postmenopausal women	Ann Intern Med
4	(42)	65	23.47	0.04	Physical therapy approaches to reduce fall and fracture risk among older adults	Nat Rev Endocrinol
5	(43)	60	31.07	0.18	Can vigorous exercise play a role in osteoporosis prevention? A review	Osteoporosis int
6	(44)	59	29.62	0.01	Exercise effects on bone mass in postmenopausal women are site-specific and load-dependent	J Bone Miner Res
7	(45)	57	28.66	0.03	Effects of resistance training on regional and total bone mineral density in premenopausal women: a randomized prospective study	J Bone Miner Res
8	(46)	53	26.6	0.02	Randomized controlled trial of effect of high-impact exercise on selected risk factors for osteoporotic fractures	Lancet
9	(47)	52	27.23	0	The Effect of Exercise Training Programs on Bone Mass: A Meta analysis of Published Controlled Trials in Pre- and Postmenopausal Women	Osteoporosis Int
10	(48)	50	18.72	0.2	Interventions for preventing falls in older people living in the community.	Cochrane Database Syst Rev

### Analysis of keywords

**Figure 7:**
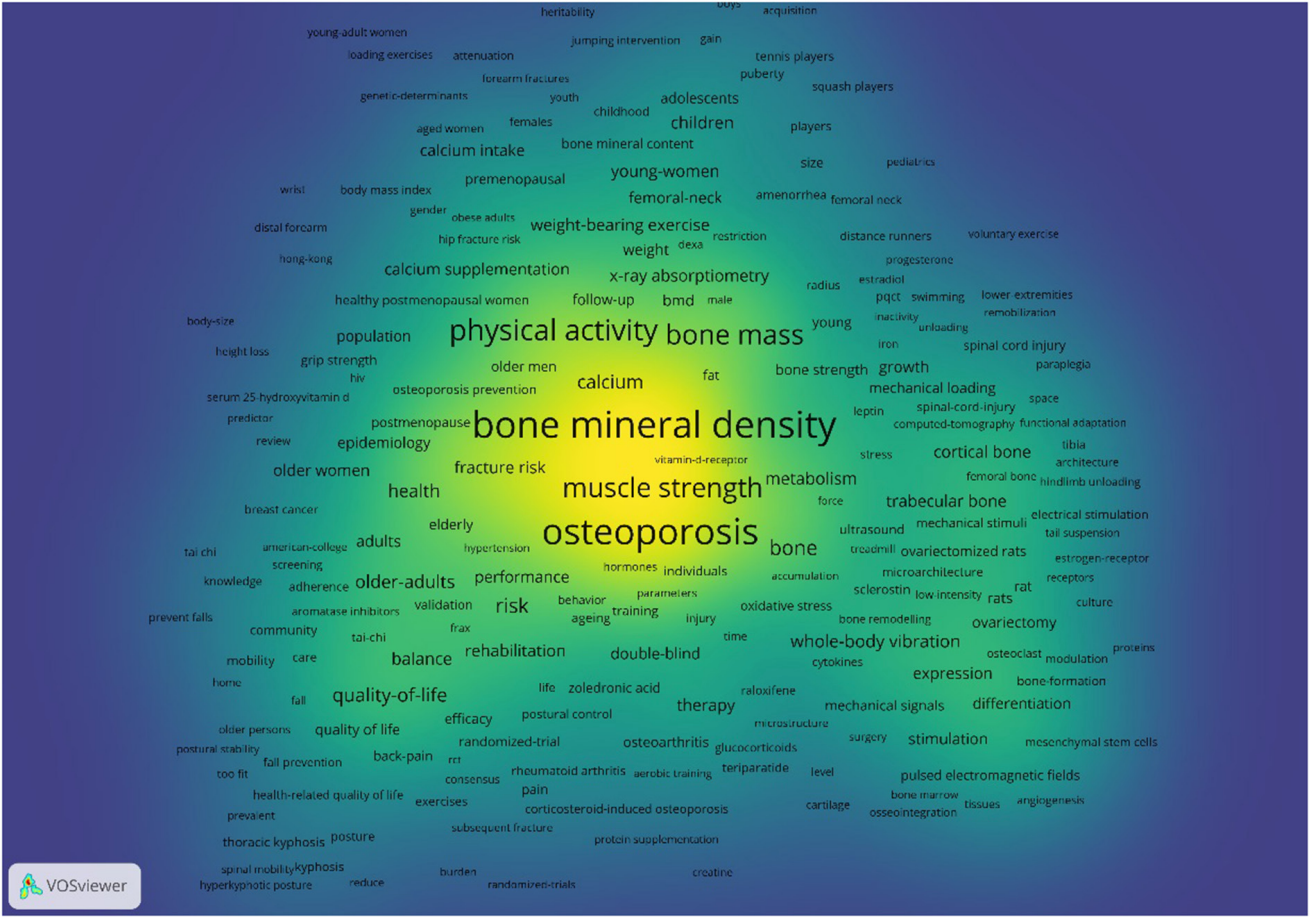
Co-occurrence analysis of keywords by density visualization.

**Figure 8:**
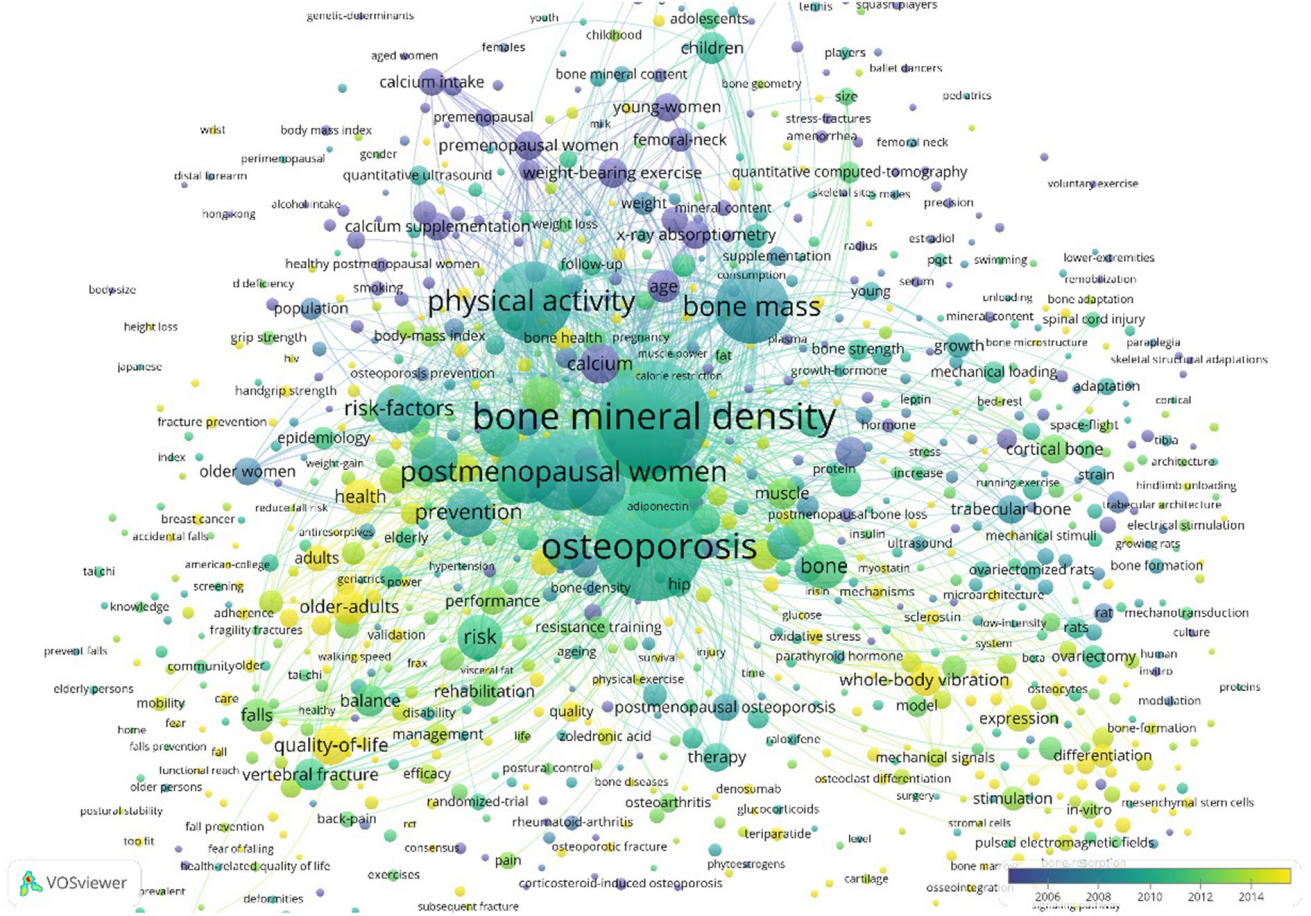
Co-occurrence analysis of keywords by overlay visualization.

When the keywords with the equal meaning were combined, the frequency of the published keywords was statistically determined and the top 30 keywords are summarized in Table 6. A map of keywords can present major objects and hot topics of research. The co-occurrence network of keywords was performed by applying VOS viewer software. We created the map based on bibliographic data with a full counting strategy, which set the minimum number of occurrences of a keyword as five. 1,062 of 7,677 keywords met the criterion after being thesaurus-cleaned. Based on keyword research categories, we incorporated VOS viewer's classification and separated keywords into three color-coded groups (Supplementary Figure S2). In this figure, purple, red, and grass green are represented for the applications of Physical Activity/ Exercise in menopausal osteoporosis, age-related osteoporosis, juvenile osteoporosis, preclinical study, osteoporosis-related fractures, respectively. These application domains would be further discussed in the next part. In addition, we also performed co-occurrence analysis of keywords by density visualization and overlay visualization in Figures 7, 8.

**Table 6:** The top 30 keywords of osteoporosis rehabilitation-related studies that were published from 1965 to 2022.

**Rank**	**Frequency**	**Centrality**	**Keyword**
1	2,024	0	Bone mineral density
2	972	0	Postmenopausal women
3	928	0.02	Physical activity
4	893	0.01	Exercise
5	827	0.02	Muscle strength
6	827	0.01	Fracture
7	814	0.02	Risk factor
8	549	0.05	Women
9	464	0.05	Randomized controlled trial
10	340	0.03	Calcium supplementation
11	338	0.04	Prevention
12	328	0.08	Bone
13	273	0.06	Men
14	260	0.08	Body composition
15	238	0.02	Quality of life
16	200	0.06	Age
17	193	0.04	Health
18	167	0.01	Older adult
19	164	0.03	Hormone replacement therapy
20	162	0.05	Cigarette smoking
21	157	0.03	Young women
22	149	0.06	Skeletal muscle
23	145	0.04	Weight bearing exercise
24	144	0.05	Estrogen
25	142	0.05	Older women
26	131	0.02	Children
27	130	0.01	Whole body vibration
28	127	0.04	Metabolism
29	126	0.04	Parathyroid hormone
30	125	0.04	Therapy

### Analysis of the burst of keywords

**Figure 9:**
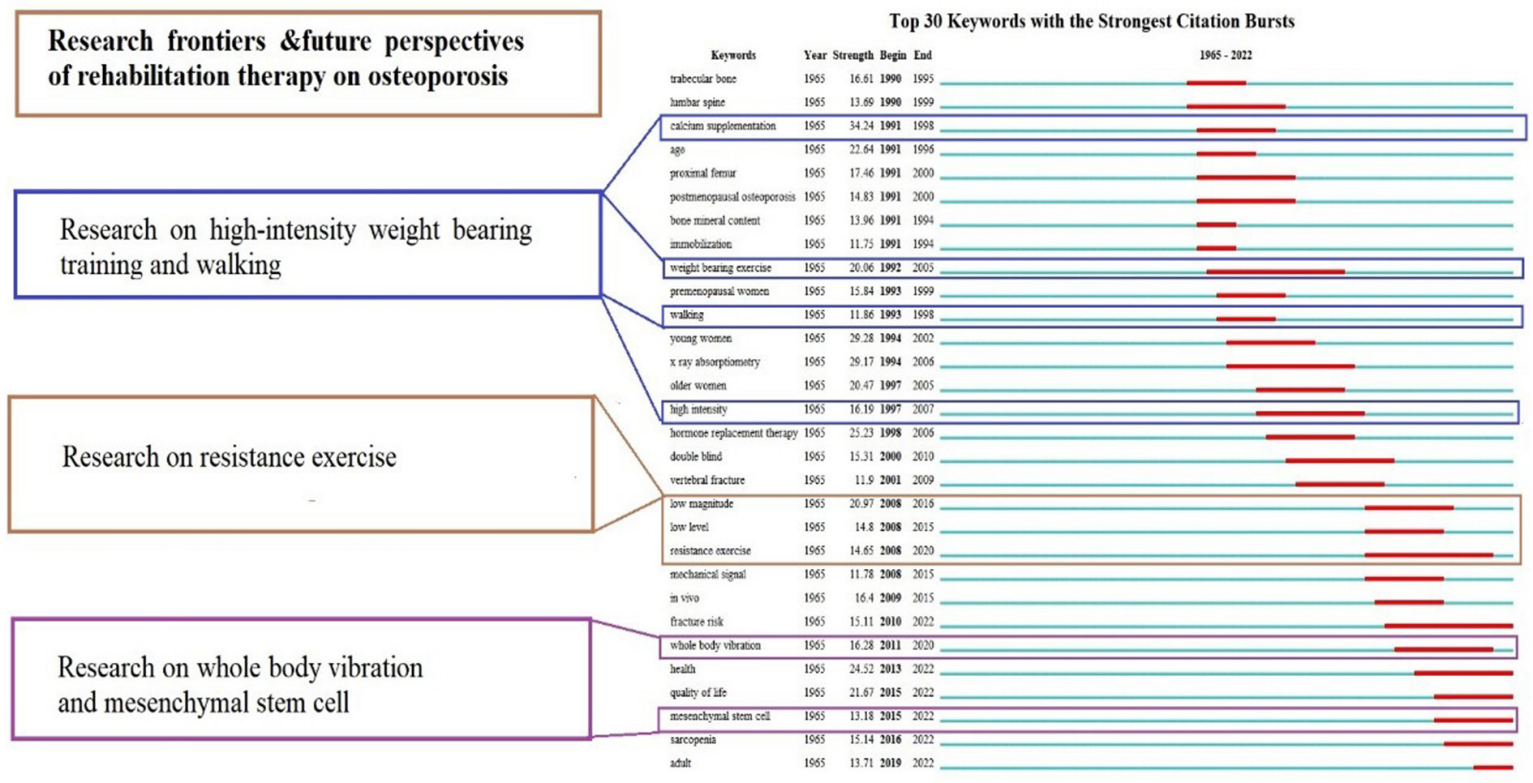
Top 30 keywords with the strongest citation burst in osteoporosis rehabilitation-related field.

The “burst words” serve as words that are cited frequently over a period of time, which can display the frontier topics and key areas of research. The top 30 keywords with the strongest citation bursts from 1965 to 2022 was shown in Figure 9. The top five keywords with the highest strength burst were calcium supplement calcium supplement (34.24), young woman (29.28), X-ray absorptiometry (29.17), hormone replacement therapy (25.23), age (22.64), Quality of Life (21.67), low magnitude (20.97), older woman (20.47), weight bearing exercise (20.06). The values in brackets follow the strength of burst, occurrence burst, The top five keywords with the most duration of burst included weight bearing exercise (1992–2005), X-ray absorptiometry (1994–2006), resistance exercise (2008–2020), fracture risk (2010–2020). During the decade of 1990–2000, Trabecular bone, lumbar spine, calcium supplement grew to be the focal point of research in this time period. From 2015 to 2022, keywords with the strongest citation bursts including fracture risk, mesenchymal stem cell, Quality of Life, sarcopenia were widely mentioned and studied.

## Discussion

### International research status of osteoporosis rehabilitation

The research presented the first bibliometric analysis of the global research trends in rehabilitation therapy for osteoporosis. Until 1991, there was a very low number of published documents per year, a sharp rise was observed in the following year. Throughout the investigated period of 31 years, the number of papers produced per year shown a gradual upward trend. Particularly, over the past decade, there has been a continual and significant growth in the number of publications published. There were 197 articles published in 2021, which is a historical peak, indicating an increase in interest in osteoporosis rehabilitation research. Several factors contribute to the rapid advancement of research on osteoporosis rehabilitation, according to available knowledge.

### Research hotspots by country (region)

The USA accounted for approximately one-fourth of the top 10 countries' documents, reflecting its strong research capacity and commitment to the advancement of the field. A noteworthy fact is that China ranked second in terms of the number of articles published. This indicates that China is placing a greater emphasis on research advancements in this subject. In addition, the USA is also followed by high-developed countries such as the UK or Australia. This is due to the fact that they have been well represented and consolidated within the World Confederation for Physical Therapy, an organization that provides support and promotes research pertaining to physical therapy (PT). Furthermore, since the USA possessed the highest degree of centrality, it also serves as the central collaborator of other countries.

### Research hotspots by institutions

Among the research institutions, University of Melbourne published the greatest number of articles, which could be attributable to universities' robust academic environments and scientific research foundations. Researchers can acquire timely information regarding osteoporosis rehabilitation research. Cooperation between institutions is mainly distributed in European and American countries based on the cooperation network of institutions. Unfortunately, despite China's large population, research on osteoporosis rehabilitation therapy is still in its infancy, and interinstitutional collaboration is lacking. Asian countries were not represented in the top 10 institutions for osteoporosis rehabilitation research, which could impediment the development of this field. Collaboration among osteoporosis rehabilitation researchers helps advance the field. Consequently, we advised strengthening collaboration networks between European, American, and Asian research institutes.

### Research hotspots by journals

The top three published journals are Osteoporosis International, Bone, and Journal of Bone and Mineral Research. Osteoporosis International journal established by the International Osteoporosis Foundation and the National Osteoporosis Foundation of the USA. It facilitates the exchange of ideas about osteoporosis and other metabolic bone diseases Journal of Bone is an unsurpassed reputation for excellence international journal that dealing with both normal and pathological processes affecting bone metabolism. The Journal of Bone and Mineral Research provides a diverse, highly impactful research with the latest insights into musculoskeletal system and mineral metabolism. It is worth noting that New England Journal of Medicine, with its impact factor of 74.699, has also published many studies on osteoporosis rehabilitation. Furthermore, four out of the 11 leading prolific publications were dedicated to the scope of rehabilitation therapy: Medicine and Science in Sports and Exercise, Sports Medicine, Scandinavian Journal of Medicine & Science in Sports, Archives of Physical Medicine and Rehabilitation. In spite of the fact that other journals such as Calcified Tissue International, Journal of Clinical Endocrinology & Metabolism are not exclusively PT-oriented, these journals have a significant advantage in our results. This may be due to osteoporosis being a multidisciplinary field, which typically provides authors with a variety of publishing alternatives, the dynamics of these journals should therefore be considered in future research.

### Research hotspots by author

Based on author contribution and co-citations, influential authors include Heinonen A, Sievanen H, Sinaki M. Cummings S and Rubin C. The researchers Heinonen A and Sievanen H, both of whom are affiliated with the UKK Institute for Health Promotion Research, have contributed 66 publications Heinonen's key academic achievements were published in the Lancet joins an increasing number of controlled trials that provide concrete evidence of the positive effects of exercise on the skeletal system a (46). According to Sievanen H, Among vitamin D-supplemented older adults living in the community, strength and balance training may be the most effective and feasible way to prevent injurious falls (49). Sinaki M is a well-known rehabilitation specialist from Mayo Clinic, she has designed a variety of exercise programs for strengthening the back muscles to reduce back pain, kyphosis, vertebral fractures, and the risk of falls. She also advocated that the Rehabilitation of Osteoporosis Program-Exercise (ROPE) incorporates complete osteoporosis management. Her research reported that exercise for patients with osteoporosis, providing recommendations for management of vertebral compression fractures and trunk strengthening for fall prevention (50). Cummings S is a famous professor from University of California San Francisco, Cummings's the major research contribution was to explore the role of various drugs such as Raloxifene, zoledronic acid, alendronate, Estrogen replacement therapy for the treatment and prevention of osteoporosis and osteoporotic fractures. Several of his articles have been published in internationally renowned magazines, including JAMA, New England Journal of Medicine, Lancet (51–56). Rubin C is from Stony Brook University and his research was focused on the effects of mechanical stimulation on bone cell and osteoporosis (57–61). He proposed that mechanical targeting of the bone marrow stem-cell pool might serve as a potentially effective, drug-free strategy of reversing the age-related decline of the musculoskeletal system (62). The authors mentioned above have published a lot of far-reaching literature on the prevention and treatment of osteoporosis and osteoporotic fractures, particularly in the area of rehabilitation for osteoporosis. It is therefore recommended that those concerned with osteoporosis pay more attention to these experts.

### Citation information

Top 10 co-cited references are listed in Table 5, The 10 important articles discovered by integrating the cited frequency and burst strength. the disciplinary framework and knowledge basis of rehabilitation studies on osteoporosis by analyzing these key papers can be gained. Dalsky et al. (41) assessed the effect of weight-bearing exercise training and subsequent detraining on lumbar bone mineral content in postmenopausal women and Nelson et al. (39) investigated the effectiveness of high-intensity strength training on multiple osteoporotic fracture risk factors. Which these two studies represented a strong research hotspot with burst strength as 41.74 and 41.27. One reviews by Howe et al. (40) focusing on the effectiveness of exercise programs in preventing bone loss and fractures in postmenopausal women published in Cochrane Database of Systematic Reviews, and exercise may be a safe and effective strategy to prevent bone loss in postmenopausal women. Karinkanta et al. (42) provided insights that they concentrate on evidence-based physical therapy approaches such as exercise, vibration training and enhancements of security at home and during periods of mobility for reducing fall and fracture risk among older adults. In recent years, Watson et al. (17) showed that High-intensity resistance and impact training (HiRIT) program improved indices of bone strength and functional performance in postmenopausal women with low bone mass. HiRIT was effective and caused no unfavorable events under highly supervised prerequisites for the healthy postmenopausal women with low to very low bone mass. Sherrington et al. (63) provided insights that exercise programs help older adults in the community experience fewer falls and at a lower rate of occurrence, exercise program to reduce falls mainly involves balance and functional exercises. Beck et al. (14) provided detailed, evidence-based guidelines for safe and effective exercise prescription for osteoporosis prevention or management.

### The hotspot and frontiers

Keyword co-occurrence and reference co-citation analysis may be performed to establish the current research focus and development trends of a specific area. Based on the overlay and density visualizations and the detection of burst keywords, we identified the research hotspots and development frontiers in osteoporosis rehabilitation-related research as discussed in the following paragraphs.

### The significance of physical activity and exercise in the prevention and management of osteoporosis

The terms physical activity encompasses leisure time physical activities (exercise and sport), daily activities, household tasks and work (64), physical activity can vary significantly in terms of both intensity and duration. Exercise refers to a planned, systematic, and repetitive physical activity that involves bodily movements with or without a specific goal for improving fitness (65). Greater levels of physical activity have frequently been related with improved health and quality of life, whereas low levels of physical activity are linked to negative health outcomes.

A variety of physical activity interventions have been designed and evaluated, and its benefits for healthy aging are well established (66). The management of osteoporosis can be improved by engaging in physical activity according to various guidelines. Some research indicated that physical activity programs probably increase bone mineral density in the lumbar spine and hip (femoral neck), and additionally improves muscle strength, balance, and joint proprioception reduce the risk of falls and fractures (67). Interestingly, physical activity and exercise seem to improve BMD both cross-sectionally and prospectively, at least appearing to exert a homeostatic effect on BMD during aging (68). Specifically, in older adults, research suggests that resistance training and weight-bearing exercise are most effective at maintaining and increasing BMD (68). Weight-bearing and resistance training can increase balance, posture, agility, and strength, which may lower the risk of falling. Moreover, some types of weight-bearing exercise may boost bone density are walking, jogging, Tai Chi, climbing stairs, and dancing. muscle-strengthening exercises usually involve weight training and other resistance exercises (9). For optimizing musculoskeletal health and function, targeted multimodal programs integrating conventional and high-velocity progressive resistance training (PRT), weight-bearing impact exercises, and challenging balance/mobility activities seemed to be the most effective (69). As reported in a meta-analysis by Martyn-St James and Carroll, that integrating jogging with low-impact exercise or trying to combine impact activities with high-intensity exercise (i.e., resistance training) is effective for maintaining BMD in postmenopausal women (70). A systematic review also revealed that multicomponent training (MT) comprising of strength, aerobic, high-impact, and weight-bearing exercises can improve or at the very least slow the age-related loss of bone mass (71). However, it is not clear which MT method will achieve better results. Therefore, further research is required to determine the appropriate multicomponent training regimens.

Furthermore, whole body vibration (WBV) training is regarded relatively safe, and does not require a high level of motivation for its practice therefore, it may be presumed as an adjunctive therapy to counteract BMD loss, particularly for those with barriers in the practice of high-impact physical exercise. Some meta-analysis and RCT demonstrated WBV significantly increased bone density (21, 72). WBV may be a practical and effective strategy to decrease well-recognized risk factors for fractures and falls, as well as to enhance balance and some aspects of neuromuscular function (73). However, parameters used in WBV (frequency, magnitude, cumulative dose, positioning on the oscillation board, and type of vibration) are extremely heterogeneous, which makes it difficult for studies to be compared. Furthermore, there has been a lack of high-quality trials showing positive associations so far. To address the knowledge gap regarding the osteogenic effects of WBV and the underlying processes in regulating osteoporosis treatment, future research should assess the effects of various WBV training protocols on bone health.

### Enhancing the management of physical activity/ exercise on osteoporosis in adolescents and men

Postmenopausal and senile osteoporosis are the two most common types of osteoporosis, accordingly, more investigation has been conducted on these two forms of osteoporosis. It is worth noting that a growing body of research has been conducted on osteoporosis in adolescents and men in recent years as well. There are a variety of strategies for combating osteoporosis, with the optimization of peak bone mass (PBM) throughout childhood being one of the most frequently utilized preventive therapy (74). PBM is a critical determinant of osteoporotic fracture risk. It has been proven that when PBM increases by 10% in children and adolescents, the risk of osteoporotic fracture was reduced by 50%, and when BMD increases by 5%, the risk will decrease by 40% (75, 76). Multiple studies have demonstrated that premenarche, and even prepubertal vs. early pubertal, are stages of greater bone response to exercise relative to postmenarche (77–80). Therefore, childhood and adolescence are pivotal periods to adopt lifestyle interventions that may prevent osteopenia and osteoporosis-related fractures in later stages of life. Jumping exercises during childhood and adolescence enhance bone density, mineral content, and structural characteristics without causing any adverse outcomes. To enhance bone mass during early childhood these types of measures should thus be employed (81). Recent systematic reviews found weight-bearing activities in childhood and adolescence can dramatically increase bone mineral content (BMC) and bone mineral density (BMD) (82).

Research on osteoporosis and physical activity commonly focused on women, male osteoporosis is still underrecognized and underdiagnosed, which logically leads to a lack of therapy options for men. The quality of life (HRQoL) of these patients may be impaired by the inadequate management of osteoporosis in males. A recent systematic review and meta-analysis revealed that males with osteoporosis had a decreased HRQoL than men without the condition, with hip fracture, vertebral fractures, or wrist fractures dramatically reducing HRQoL of men. Importantly, BMD at the spine and femur was strongly associated with HRQoL (83). Research showed that higher BMD was maintained in older males who engaged in high-impact, quick-impact physical activity, and a lower risk of falling was associated with higher energy expenditure (84). However, some study indicated that physical activity has almost no effect on BMD at the total hip and little or no effect on BMD at the femoral neck, lumbar spine, and whole body (85). The main reasons for inconsistent study results may be the diversity of the interventions, the small sample size, and the (relatively) short duration of the interventions. To improve the quality of the evidence, future research should enlarge sample size, identify interventions, engage in comprehensive reporting of HRQoL outcomes and prolong long-term interventions.

### The key role of physical activity and exercise in the prevention and management of osteoporosis-related sarcopenia and fractures

Osteosarcopenia was first established by Duque and coworkers to represent a subpopulation of elderly individuals impacted by osteoporosis and sarcopenia (86) osteosarcopenia is a unique syndrome characterized by poor bone density and decreased muscle mass, strength, and/or functional capacity (87). The researchers revealed that osteosarcopenia dramatically raised the likelihood of fractures, falls, and mortality (88). Compared to those without osteosarcopenia, with sarcopenia alone, and those with osteoporosis alone, women with osteosarcopenia were more likely to have previously suffered a fracture (89). Therefore, the elderly people should be mindful of the risks associated with osteosarcopenia. The researchers investigated the efficacy of non-pharmacological (exercise and/or dietary) therapies on musculoskeletal measures and outcomes in osteosarcopenic individuals and found that RT can boost gains in muscle mass, strength, and quality, as well as increase or maintain BMD in older osteosarcopenic adults (90). A study by Kemmler also showed that high intensity dynamic resistance exercise (HIT-DRT) on devices especially when combined with moderate protein supplementation was proven to be a safe, appealing, highly efficient and effective strategy for managing osteosarcopenia in older men with sarcopenia and osteoporosis (91). As far as muscle quality (MQ)parameters are concerned, intermittent exercise regimens with intervals of 6 months or longer should be substituted with largely continuous regimens (92). Consequently, suitable therapy routes involving moderate-to-high intensity supervised RT should be promoted in clinical settings for individuals with osteosarcopenia. For osteosarcopenia upstream prevention, current recommendations should emphasize large-scale RT programs that are aimed at increasing participation of older adults. These strategies may ultimately lessen the socioeconomic impact of this geriatric disease.

Bone fragility caused by osteoporosis is common in older persons and is associated with a higher of fragility fracture. However, osteoporosis may not be detected until the patient has suffered multiple fragility fractures. It is estimated that 319 million adults aged 50 years or more will be at significantly higher risk for osteoporotic fractures worldwide (93). Individuals who suffer fragility fractures are at higher risk of developing subsequent fracture at a different site. Based on a meta-analysis conducted by Kanis and colleagues, prior fractures are associated with an 86% increased risk of further fracture at any new site (94). Therefore, The International Osteoporosis Foundation offers a best practice suggestion for secondary fracture prevention, emphasizing the need to diagnose patients early, identify their future fracture risk, evaluate the patient in a reasonable timeframe, and then determine the next measure. Exercise approach is usually advised for persons who are deemed to be at low or moderate risk for osteoporotic fractures (95). According to the study, multimodal exercise can reduce the risk of falling in participants at high risk of primary osteoporotic fractures when compared with a control group (96). PRT can enhances physical function, life quality, and relieves pain. Current research suggests that PRT should be prescribed for those at risk of fracture, however determining the optimal frequency or intensity of PRT is difficult due to its heterogeneity and limitations. A hip protector is one of the many multifactorial interventions that can be used to prevent fall and fractures among patients living in high-risk residential settings. A systematic review found moderate quality evidence supporting a small reduction in hip fracture risk (97). Generally, Fractures of conservative treatment indicated the benefits of early initiation of exercise therapy and physiotherapy. Nevertheless, reliable data on the optimal duration and intensity of physiotherapy and use of orthoses remain lacking (98). A sufficient number of studies have not been conducted to determine whether exercise affects incident fractures, falls, or adverse events. High-quality randomized trials are required in the future to determine the safety and efficacy of exercise in reducing the incidence of fractures and falls, improving patient-centered outcomes (pain, function) in individuals with fractures (99).

### Application of mesenchymal stem cells (MSCs) in the prevention and treatment of osteoporosis

During osteoporosis pathogenesis and treatment, MSCs play a vital role due to their multi-directional differentiation potential and self-renewal ability (100). On the one hand, a number of factors contribute to the pathogenesis of osteoporosis, including homing disorders, impaired osteogenic differentiation, senescence of MSCs, an imbalanced microenvironment (such as transcription factors, signal pathways, and microRNAs), and disrupted immunoregulation (101). On the other hand, a number of preclinical studies have shown that MSC transplantation can enhance osteogenic differentiation, increase bone mineral density, and slow down osteoporosis progression (101). The latest techniques, such as gene modification (overexpressing osteogenic and angiogenic genes, knocking down bone destruction genes, modifying homing-related genes, causing MSCs to delay senescence), targeted modification, and co-transplantation, offer promising results for enhancing MSC therapeutic effectiveness (102). Despite this, it remains unclear which regulatory mechanisms and molecular markers can be used to assess MSC migration to the bone surface, which is crucial for bone formation and fracture healing. As a result, it is difficult to regulate MSC activity when managing osteoporosis and fractures. In addition, due to some safety concerns, the effectiveness of transplantation, and uniformity of manufacturing processes, no pertinent clinical study data have yet been reported in terms of clinical trial investigations on MSC for osteoporosis. Therefore, future studies ought to investigate the regulatory mechanisms of MSCs in the management of osteoporosis, and research should concentrate on the effectiveness of readily available and highly biocompatible autologous adipose stem cells in the osteoporosis therapy.

### Strengths and limitations

Based on bibliometric and visual analyses, this study examined the progress and trends in global scientific research into osteoporosis rehabilitation therapy. With the CiteSpace and VOSview software, a comprehensive analysis and a visual literature network for co-occurrence and co-citation were performed. Moreover, researchers are now able to quickly grasp the present state of investigation, hot topics, and development trends in this area through visualization of the top ten references with the strongest citation bursts. In spite of this, limitations are unavoidable. First, we put a lot of emphasis exclusively on publications from solely WoS core databases, consequently, several crucial studies may be excluded. It would have been preferable to combine the findings with those from other databases (such as PubMed and Scopus). Notably, Web of Science is the most widely used database in scientometrics, and the majority of bibliometric tools can detect data from this database. Second, there was limited coverage of literature in the field of osteoporosis rehabilitation because only articles and reviews of English-language publications were included, which may have resulted in the omission of high-quality literature in other languages. Therefore, publication bias may be caused by these factors. Additionally, as osteoporosis rehabilitation has been a developing research field in recent years, some recently published, high-quality papers may have a low citation frequency due to their short publication time. There is therefore a discrepancy between the research results and the actual situation. Last but not least, the bibliometric analysis is only a tool, and the results may vary from what you see in real-world research.

## Conclusion

During the last few years, the number of published studies on the rehabilitation of osteoporosis has increased significantly. Osteoporosis rehabilitation therapy will continue to receive an increasing number of publications. In conclusion, the study presented the first bibliometric and visual analysis of international research on osteoporosis rehabilitation utilizing the Web of Science database, CiteSpace and VOS view software, and displayed a relatively scientific and intuitive overview of osteoporosis rehabilitation research. We rigorously evaluated publishing data pertaining to the number of published articles, influential nations and institutions, authors and co-cited authors, published journals, and collaborative networks. In addition, we presented both historical and prospective insights into osteoporosis rehabilitation strategies, as well as information regarding major research hotspots, development trends, and frontiers. At present, the major modes and parameters of physical activity/exercise for osteoporosis (including WBV, weight bearing exercises, resistance training), targeted multicomponent training regimens, rehabilitation therapy for postmenopausal women, older women, children and men, osteoporosis related -sarcopenia and fractures, and mesenchymal stem cells are among the frontiers and hotspots of research. These areas indicate the development trend for future research and can serve as a guideline for future research. In summary, our findings may provide useful resources to scholars for understanding the present state and trend of studies on osteoporosis rehabilitation and providing references and suggestions for future research in this area.

## Data availability statement

The original contributions presented in the study are included in the article/Supplementary material, further inquiries can be directed to the corresponding authors.

## Author contributions

CH and SL: designing this study. LW: writing initial draft and revision. YLi and JH: reviewing and analyzing the literature. JJ and RW: rechecking the manuscript and providing suggestions for amendment. YLia: revising language and content. All authors contributed to the article and approved the submitted version.

## Funding

This work was supported by National Natural Science Foundation (No. 81902287), Cooperative Development Project Fund of West China Hospital, Sichuan University (No. hxh2107188), and Sichuan Provincial Department of Science and Technology (No. 2021YFS0239).

## Conflict of interest

The authors declare that the research was conducted in the absence of any commercial or financial relationships that could be construed as a potential conflict of interest.

## Publisher's note

All claims expressed in this article are solely those of the authors and do not necessarily represent those of their affiliated organizations, or those of the publisher, the editors and the reviewers. Any product that may be evaluated in this article, or claim that may be made by its manufacturer, is not guaranteed or endorsed by the publisher.

## References

[1] Romosozumab for osteoporosis. Aust Prescr. (2021) 44:109–10. 10.18773/austprescr.2021.021 34211250 PMC8236877

[2] De Vincentis A Behr AU Bellelli G Bravi M Castaldo A Cricelli C. Management of hip fracture in the older people: rationale and design of the Italian consensus on the orthogeriatric co-management. Aging Clin Exp Res. (2020) 32:1393–9. 10.1007/s40520-020-01574-4 32358728

[3] Iolascon G Moretti A Giamattei MT Migliaccio S Gimigliano F. Prevalent fragility fractures as risk factor for skeletal muscle function deficit and dysmobility syndrome in post-menopausal women. Aging Clin Exp Res. (2015) 27 Suppl 1:S11–6. 10.1007/s40520-015-0417-1 26204997

[4] Global regional and national incidence, prevalence, and years lived with disability for 301 acute and chronic diseases and injuries in 188 countries 1990-2013: 1990-2013: a systematic analysis for the Global Burden of Disease Study 2013. Lancet (London, England). (2015) 386:743–800. 26063472 10.1016/S0140-6736(15)60692-4 PMC4561509

[5] Wright NC Looker AC Saag KG Curtis JR Delzell ES Randall S. The recent prevalence of osteoporosis and low bone mass in the United States based on bone mineral density at the femoral neck or lumbar spine. J Bone Miner Res. (2014) 29:2520–6. 10.1002/jbmr.2269 24771492 PMC4757905

[6] Ayub N Faraj M Ghatan S Reijers JAA Napoli N Oei L. The treatment gap in osteoporosis. J Clin Med. (2021) 10:10133002. 10.3390/jcm10133002 34279485 PMC8268346

[7] Weycker D Li X Barron R Bornheimer R Chandler D. Hospitalizations for osteoporosis-related fractures: economic costs and clinical outcomes. Bone Rep. (2016) 5:186–91. 10.1016/j.bonr.2016.07.005 28580386 PMC5440958

[8] Ivanova S Vasileva L. Current and emerging strategies in osteoporosis management. Curr Pharm Des. (2017) 23:6279–87. 10.2174/1381612823666170714122714 28714404

[9] Camacho PM Petak SM Binkley N Diab DL Eldeiry LS Farooki A. American Association of Clinical Endocrinologists/American College of Endocrinology Clinical Practice Guidelines for the Diagnosis and Treatment of Postmenopausal Osteoporosis-−2020 Update. Endocrine Pract. (2020) 26:1–46. 10.4158/GL-2020-0524SUPPL 32427503

[10] Gregson CL Armstrong DJ Bowden J Cooper C Edwards J Gittoes NJL. UK clinical guideline for the prevention and treatment of osteoporosis. Arch Osteoporos. (2022) 17:58. 10.1007/s11657-022-01061-5 35378630 PMC8979902

[11] Osteoporosis prevention S and diagnosis: ACOG clinical practice guideline No. 1. Obstetrics Gynecol. (2021) 138:494–506. 10.1097/AOG.0000000000004514 34412075

[12] Meeta M Harinarayan CV Marwah R Sahay R Kalra S Babhulkar S. Clinical practice guidelines on postmenopausal osteoporosis: ^*^an executive summary and recommendations–update 2019-2020. J Midlife Health. (2020) 11:96–112. 10.4103/jmh.JMH_143_20 33281419 PMC7688018

[13] Hartley GW Roach KE Nithman RW Betz SR Lindsey C Fuchs RK. Physical therapist management of patients with suspected or confirmed osteoporosis: a clinical practice guideline from the academy of geriatric physical therapy. J Geriatr Phys Ther. (2001) 2022:E106–19. 10.1519/JPT.0000000000000346 35384943 PMC8983944

[14] Beck BR Daly RM Singh MA Taaffe DR. Exercise and Sports Science Australia (ESSA) position statement on exercise prescription for the prevention and management of osteoporosis. J Sci Med Sport. (2017) 20:438–45. 10.1016/j.jsams.2016.10.001 27840033

[15] Iolascon G de Sire A Curci C Paoletta M Liguori S Calafiore D. Osteoporosis guidelines from a rehabilitation perspective: systematic analysis and quality appraisal using AGREE II. Eur J Phys Rehabil Med. (2021) 57:273–9. 10.23736/S1973-9087.21.06581-3 33650841

[16] ElDeeb AM Abdel-Aziem AA. Effect of whole-body vibration exercise on power profile and bone mineral density in postmenopausal women with osteoporosis: a randomized controlled trial. J Manipulative Physiol Ther. (2020) 43:384–93. 10.1016/j.jmpt.2019.12.003 32868028

[17] Watson SL Weeks BK Weis LJ Harding AT Horan SA Beck BR. High-intensity resistance and impact training improves bone mineral density and physical function in postmenopausal women with osteopenia and osteoporosis: the LIFTMOR randomized controlled trial. J Bone Miner Res. (2018) 33:211–20. 10.1002/jbmr.3284 28975661

[18] Stanghelle B Bentzen H Giangregorio L Pripp AH Skelton DA Bergland A. Effects of a resistance and balance exercise programme on physical fitness, health-related quality of life and fear of falling in older women with osteoporosis and vertebral fracture: a randomized controlled trial. Osteoporosis. (2020) 31:1069–78. 10.1007/s00198-019-05256-4 31925473

[19] Anupama DS Norohna JA Acharya KK. Ravishankar, George A. Effect of exercise on bone mineral density and quality of life among postmenopausal women with osteoporosis without fracture: a systematic review. Int J Orthop Trauma Nurs. (2020) 39:100796. 10.1016/j.ijotn.2020.100796 33041224

[20] Rodrigues IB Ponzano M Hosseini Z Thabane L Chilibeck PD Butt DA. The Effect of impact exercise (alone or multicomponent intervention) on health-related outcomes in individuals at risk of fractures: a systematic review and meta-analysis of randomized controlled trials. Sports Med (Auckland, NZ). (2021) 51:1273–92. 10.1007/s40279-021-01432-x 33914282

[21] DadeMatthews OO Agostinelli PJ Neal FK Oladipupo SO Hirschhorn RM Wilson AE. Systematic review and meta-analyses on the effects of whole-body vibration on bone health. Complement Ther Med. (2022) 65:102811. 10.1016/j.ctim.2022.102811 35093509

[22] Ponzano M Rodrigues IB Hosseini Z Ashe MC Butt DA Chilibeck PD. Progressive resistance training for improving health-related outcomes in people at risk of fracture: a systematic review and meta-analysis of randomized controlled trials. Phy Ther. (2021) 101:221. 10.1093/ptj/pzaa221 33367736

[23] Yan Y Tan B Fu F Chen Q Li W Chen W. Exercise vs conventional treatment for treatment of primary osteoporosis: a systematic review and meta-analysis of randomized controlled trials. Orthop Surg. (2021) 13:1474–87. 10.1111/os.13036 34124845 PMC8313149

[24] Ellegaard O Wallin JA. The bibliometric analysis of scholarly production: How great is the impact? Scientometrics. (2015) 105:1809–31. 10.1007/s11192-015-1645-z 26594073 PMC4643120

[25] Aydinoglu AU Taşkin Z. Origins of life research: a bibliometric approach. Orig Life Evol Biosph. (2018) 48:55–71. 10.1007/s11084-017-9543-4 28702783

[26] Leclerc A Chastang JF Kaniewski N Cyr D Ozguler A Descatha A. The bibliographic impact of epidemiological studies: what can be learnt from citations? Occup Environ Med. (2010) 67:213–6. 10.1136/oem.2009.046425 19819856 PMC2874724

[27] Li B Hu K Lysenko V Khan KY Wang Y Jiang Y. A scientometric analysis of agricultural pollution by using bibliometric software VoSViewer and Histcite™. Environ Sci Pollut Res Int. (2022) 29:37882–93. 10.1007/s11356-022-18491-w 35067891

[28] Huang YJ Cheng S Yang FQ Chen C. Analysis and visualization of research on resilient cities and communities based on VOSviewer. Int J Environ Res Public Health. (2022) 19:68. 10.3390/ijerph19127068 35742316 PMC9223032

[29] Oyewola DO Dada EG. Exploring machine learning: a scientometrics approach using bibliometrix and VOSviewer. SN Appl Sci. (2022) 4:143. 10.1007/s42452-022-05027-7 35434524 PMC8996204

[30] Chen Y Lin M Zhuang D. Wastewater treatment and emerging contaminants: Bibliometric analysis. Chemosphere. (2022) 297:133932. 10.1016/j.chemosphere.2022.133932 35149018

[31] Shen J Shen H Ke L Chen J Dang X Liu B. Knowledge mapping of immunotherapy for hepatocellular carcinoma: a bibliometric study. Front Immunol. (2022) 13:815575. 10.3389/fimmu.2022.815575 35173728 PMC8841606

[32] Wu H Sun Z Tong L Wang Y Yan H Sun Z. Bibliometric analysis of global research trends on male osteoporosis: a neglected field deserves more attention. Arch Osteoporos. (2021) 16:154. 10.1007/s11657-021-01016-2 34632530

[33] Yun L Wang L Pan Y Liu M Han Q Sun J. Current status and development trend of miRNAs in osteoporosis-related research: a bibliometric analysis. Folia histochemica et cytobiologica. (2021) 59:203–11. 10.5603/FHC.a2021.0024 34852180

[34] Zhang Y Hu M Zhu L Wang J Wang P Shi P. Bisphosphates for osteoporosis: a bibliometric analysis of the most cited articles. eCAM. (2022) 2022:4565069. 10.1155/2022/4565069 35646145 PMC9132659

[35] Yan W Zheng K Weng L Chen C Kiartivich S Jiang X. Bibliometric evaluation of 2000-2019 publications on functional near-infrared spectroscopy. Neuroimage. (2020) 220:117121. 10.1016/j.neuroimage.2020.117121 32619709

[36] Lu C Li X Yang K. Trends in shared decision-making studies from 2009 to 2018: a bibliometric analysis. Front Public Health. (2019) 7:384. 10.3389/fpubh.2019.00384 31921749 PMC6930165

[37] Synnestvedt MB Chen C Holmes JH. CiteSpace II: visualization and knowledge discovery in bibliographic databases. AMIA. (2005) 2005:724–8. 16779135 PMC1560567

[38] Liang C Luo A Zhong Z. Knowledge mapping of medication literacy study: a visualized analysis using CiteSpace. SAGE Open Med. (2018) 6:2050312118800199. 10.1177/2050312118800199 30245817 PMC6144508

[39] Nelson ME Fiatarone MA Morganti CM Trice I Greenberg RA Evans WJ. Effects of high-intensity strength training on multiple risk factors for osteoporotic fractures. A randomized controlled trial. JAMA. (1994) 272:1909–14. 10.1001/jama.272.24.1909 7990242

[40] Howe TE Shea B Dawson LJ Downie F Murray A Ross C. Exercise for preventing and treating osteoporosis in postmenopausal women. Cochrane Database Syst Rev. (2011) 7:Cd000333. 10.1002/14651858.CD000333.pub2 21735380 PMC12744941

[41] Dalsky GP Stocke KS Ehsani AA Slatopolsky E Lee WC Birge SJ Jr.. Weight-bearing exercise training and lumbar bone mineral content in postmenopausal women. Ann Intern Med. (1988) 108:824–8. 10.7326/0003-4819-108-6-824 3259410

[42] Karinkanta S Piirtola M Sievänen H Uusi-Rasi K Kannus P. Physical therapy approaches to reduce fall and fracture risk among older adults. Nat Rev Endocrinol. (2010) 6:396–407. 10.1038/nrendo.2010.70 20517287

[43] Gutin B Kasper MJ. Can vigorous exercise play a role in osteoporosis prevention? A review. Osteoporos Int. (1992) 2:55–69. 10.1007/BF01623838 1536981

[44] Kerr D Morton A Dick I Prince R. Exercise effects on bone mass in postmenopausal women are site-specific and load-dependent. J Bone Miner Res. (1996) 11:218–25. 10.1002/jbmr.5650110211 8822346

[45] Lohman T Going S Pamenter R. Effects of resistance training on regional and total bone mineral density in premenopausal women: a randomized prospective study. J Bone Miner Res. (1995) 10:1015–24. 10.1002/jbmr.5650100705 7484276

[46] Heinonen A Kannus P Sievänen H Oja P Pasanen M Rinne M. Randomised controlled trial of effect of high-impact exercise on selected risk factors for osteoporotic fractures. Lancet (London, England). (1996) 348:1343–7. 10.1016/S0140-6736(96)04214-6 8918277

[47] Wolff I Van Croonenborg JJ Kemper HC. The effect of exercise training programs on bone mass: a meta-analysis of published controlled trials in pre- and postmenopausal women. Osteoporos Int. (1999). 9:1–12. 10.1007/s001980050109 10367023

[48] Gillespie LD Robertson MC Gillespie WJ. Interventions for preventing falls in older people living in the community. Cochrane Database Syst Rev. (2012) 2012:Cd007146. 10.1002/14651858.CD007146.pub2 22972103 PMC8095069

[49] Uusi-Rasi K Patil R Karinkanta S Kannus P Tokola K Lamberg-Allardt C. Exercise and vitamin D in fall prevention among older women: a randomized clinical trial. JAMA Intern Med. (2015) 175:703–11. 10.1001/jamainternmed.2015.0225 25799402

[50] Sinaki M. Exercise for patients with osteoporosis: management of vertebral compression fractures and trunk strengthening for fall prevention. PM R. (2012) 4:882–8. 10.1016/j.pmrj.2012.10.008 23174554

[51] Whooley MA Grady D Cummings SR. Postmenopausal hormone therapy and mortality. N Engl J Med. (1997) 337:1389–90. 10.1056/NEJM199711063371913 9380092

[52] Cummings SR Melton LJ. Epidemiology and outcomes of osteoporotic fractures. Lancet (London, England). (2002) 359:1761–7. 10.1016/S0140-6736(02)08657-9 12049882

[53] Cummings SR San Martin J McClung MR Siris ES Eastell R Reid IR. Denosumab for prevention of fractures in postmenopausal women with osteoporosis. N Engl J Med. (2009) 361:756–65. 10.1056/NEJMoa0809493 19671655

[54] Black DM Cummings SR Karpf DB Cauley JA Thompson DE Nevitt MC. Randomised trial of effect of alendronate on risk of fracture in women with existing vertebral fractures. fracture intervention trial research group. Lancet (London, England). (1996) 348:1535–41. 10.1016/S0140-6736(96)07088-2 8950879

[55] Black DM Delmas PD Eastell R Reid IR Boonen S Cauley JA. Once-yearly zoledronic acid for treatment of postmenopausal osteoporosis. N Engl J Med. (2007) 356:1809–22. 10.1056/NEJMoa067312 17476007

[56] Ettinger B Black DM Mitlak BH Knickerbocker RK Nickelsen T Genant HK. Reduction of vertebral fracture risk in postmenopausal women with osteoporosis treated with raloxifene: results from a 3-year randomized clinical trial. Multiple Outcomes of Raloxifene Evaluation (MORE) Investigators. JAMA. (1999) 282:637–45. 10.1001/jama.282.7.637 10517716

[57] Patel VS Ete Chan M Rubin J Rubin CT. Marrow adiposity and hematopoiesis in aging and obesity: exercise as an intervention. Curr Osteoporos Rep. (2018) 16:105–15. 10.1007/s11914-018-0424-1 29476393 PMC5866776

[58] Judex S Rubin CT. Is bone formation induced by high-frequency mechanical signals modulated by muscle activity? J Musculoskelet Neuronal Interact. (2010) 10:3–11. 10.1016/S0140-6736(15)60692-4 20190375 PMC2919567

[59] Krishnamoorthy D Frechette DM Adler BJ Green DE Chan ME Rubin CT. Marrow adipogenesis and bone loss that parallels estrogen deficiency is slowed by low-intensity mechanical signals. Osteoporosis. (2016) 27:747–56. 10.1007/s00198-015-3289-5 26323329

[60] Kiel DP Hannan MT Barton BA Bouxsein ML Sisson E Lang T. Low-Magnitude Mechanical Stimulation To Improve Bone Density In Persons Of Advanced Age: A Randomized, Placebo-Controlled Trial. J Bone Miner Res. (2015) 30:1319–28. 10.1002/jbmr.2448 25581217 PMC4834704

[61] Chan ME Uzer G Rubin CT. The potential benefits and inherent risks of vibration as a non-drug therapy for the prevention and treatment of osteoporosis. Curr Osteoporos Rep. (2013) 11:36–44. 10.1007/s11914-012-0132-1 23371467 PMC3586310

[62] Ozcivici E Luu YK Adler B Qin YX Rubin J Judex S. Mechanical signals as anabolic agents in bone. Nat Rev Rheumatol. (2010) 6:50–9. 10.1038/nrrheum.2009.239 20046206 PMC3743048

[63] Sherrington C Fairhall NJ Wallbank GK Tiedemann A Michaleff ZA Howard K. Exercise for preventing falls in older people living in the community. Cochrane Database Syst Rev. (2019) 1:Cd012424. 10.1002/14651858.CD012424.pub2 30703272 PMC6360922

[64] Caspersen CJ Powell KE Christenson GM. Physical activity, exercise, and physical fitness: definitions and distinctions for health-related research. Public Health Rep (Washington, DC: 1974). (1985) 100:126–31. 3920711 PMC1424733

[65] American College of Sports Medicine Position Stand. Med Sci Sports Exerc. (1998) 30:992–1008. 10.1097/00005768-199806000-00033 9624662

[66] Das P Horton R. Physical activity-time to take it seriously and regularly. Lancet (London, England). (2016) 388:1254–5. 10.1016/S0140-6736(16)31070-4 27475269

[67] Chang CF Lee JI Huang SP Geng JH Chen SC. Regular exercise decreases the risk of osteoporosis in postmenopausal women. Front Public Health. (2022) 10:897363. 10.3389/fpubh.2022.897363 35784236 PMC9240347

[68] McMillan LB Zengin A Ebeling PR Scott D. Prescribing physical activity for the prevention and treatment of osteoporosis in older adults. Healthcare (Basel, Switzerland). (2017) 5:85. 10.3390/healthcare5040085 29113119 PMC5746719

[69] Daly RM. Exercise and nutritional approaches to prevent frail bones, falls and fractures: an update. Climacteric. (2017) 20:119–24. 10.1080/13697137.2017.1286890 28286988

[70] Martyn-St James M Carroll S A. meta-analysis of impact exercise on postmenopausal bone loss: the case for mixed loading exercise programmes. Br J Sports Med. (2009) 43:898–908. 10.1136/bjsm.2008.052704 18981037

[71] Gómez-Cabello A Ara I González-Agüero A Casajús JA Vicente-Rodríguez G. Effects of training on bone mass in older adults: a systematic review. Sports Med (Auckland, NZ). (2012) 42:301–25. 10.2165/11597670-000000000-00000 22376192

[72] He Z Zheng J Liu S Guan Z Zhou Q Jin X. The effect of whole-body vibration in osteopenic patients after total knee arthroplasty: a randomized controlled trial. Aging Clin Exp Res. (2022) 34:1381–90. 10.1007/s40520-021-02043-2 35028919

[73] Moreira-Marconi E Dionello CF Morel DS Sá-Caputo DC Souza-Gonçalves CR Paineiras-Domingos LL. Could whole body vibration exercises influence the risk factors for fractures in women with osteoporosis? Osteoporosis Sarcopenia. (2016) 2:214–20. 10.1016/j.afos.2016.09.003 30775489 PMC6372741

[74] Vicente-Rodríguez G. How does exercise affect bone development during growth? Sports Med (Auckland, NZ). (2006) 36:561–9. 10.2165/00007256-200636070-00002 16796394

[75] van der Sluis IM de Muinck Keizer-Schrama SM. Osteoporosis in childhood: bone density of children in health and disease. JPEM. (2001) 14:817–32. 10.1515/JPEM.2001.14.7.817 11515724

[76] Goulding A Jones IE Taylor RW Manning PJ Williams SM. More broken bones: a 4-year double cohort study of young girls with and without distal forearm fractures. J Bone Miner Res. (2000) 15:2011–8. 10.1359/jbmr.2000.15.10.2011 11028455

[77] Mackelvie KJ McKay HA Khan KM Crocker PR. A school-based exercise intervention augments bone mineral accrual in early pubertal girls. J Pediatr. (2001) 139:501–8. 10.1067/mpd.2001.118190 11598595

[78] McKay HA Petit MA Schutz RW Prior JC Barr SI Khan KM. Augmented trochanteric bone mineral density after modified physical education classes: a randomized school-based exercise intervention study in prepubescent and early pubescent children. J Pediatr. (2000) 136:156–62. 10.1016/S0022-3476(00)70095-3 10657819

[79] Petit MA McKay HA MacKelvie KJ Heinonen A Khan KM Beck TJ. randomized school-based jumping intervention confers site and maturity-specific benefits on bone structural properties in girls: a hip structural analysis study. J Bone Miner Res. (2002) 17:363–72. 10.1359/jbmr.2002.17.3.363 11874228

[80] Heinonen A Sievänen H Kannus P Oja P Pasanen M Vuori I. High-impact exercise and bones of growing girls: a 9-month controlled trial. Osteoporosis. (2000) 11:1010–7. 10.1007/s001980070021 11256891

[81] Gómez-Bruton A Matute-Llorente Á González-Agüero A Casajús JA Vicente-Rodríguez G. Plyometric exercise and bone health in children and adolescents: a systematic review. World J Pediatr. (2017) 13:112–21. 10.1007/s12519-016-0076-0 28101776

[82] Behringer M Gruetzner S McCourt M Mester J. Effects of weight-bearing activities on bone mineral content and density in children and adolescents: a meta-analysis. J Bone Miner Res. (2014) 29:467–78. 10.1002/jbmr.2036 23857721

[83] Hu J Zheng W Zhao D Sun L Zhou B Liu J. Health-related quality of life in men with osteoporosis: a systematic review and meta-analysis. Endocrine. (2021) 74:270–80. 10.1007/s12020-021-02792-0 34165773

[84] Ng CA Scott D Seibel MJ Cumming RG Naganathan V Blyth FM. Higher-impact physical activity is associated with maintenance of bone mineral density but not reduced incident falls or fractures in older men: the concord health and aging in men project. J Bone Miner Res. (2021) 36:662–72. 10.1002/jbmr.4228 33278306

[85] Ashe MC Santos IKD Edward NY Burnett LA Barnes R Fleig L. Physical activity and bone health in men: a systematic review and meta-analysis. J Bone Metab. (2021) 28:27–39. 10.11005/jbm.2021.28.1.27 33730781 PMC7973404

[86] Hirschfeld HP Kinsella R Duque G. Osteosarcopenia: where bone, muscle, and fat collide. Osteoporosis. (2017) 28:2781–90. 10.1007/s00198-017-4151-8 28733716

[87] Kirk B Al Saedi A Duque G. Osteosarcopenia: a case of geroscience. Aging Med [Milton (NSW)]. (2019) 2:147–56. 10.1002/agm2.12080 31942528 PMC6880711

[88] Teng Z Zhu Y Teng Y Long Q Hao Q Yu X. The analysis of osteosarcopenia as a risk factor for fractures, mortality, and falls. Osteoporosis. (2021) 32:2173–83. 10.1007/s00198-021-05963-x 33877382

[89] Lin YH Shih YT Teng MMH. The impact of the “Osteo” component of osteosarcopenia on fragility fractures in post-menopausal women. Int J Mol Sci. (2021) 22:5256. 10.3390/ijms22105256 34067582 PMC8155869

[90] AtlihanRKirkBDuqueG. Non-Pharmacological interventions in osteosarcopenia: a systematic review. J Nutr Health Aging. (2021) 25:25–32. 10.1007/s12603-020-1537-733367459

[91] Kemmler W Kohl M Jakob F Engelke K von Stengel S. Effects of high intensity dynamic resistance exercise and whey protein supplements on osteosarcopenia in older men with low bone and muscle mass. final results of the randomized controlled FrOST study. Nutrients. (2020) 12:e12082341. 10.3390/nu12082341 32764397 PMC7468852

[92] Ghasemikaram M Engelke K Kohl M von Stengel S Kemmler W. Detraining effects on muscle quality in older men with osteosarcopenia follow-up of the randomized controlled Franconian Osteopenia and Sarcopenia Trial (FrOST). Nutrients. (2021) 13:528. 10.3390/nu13051528 34062828 PMC8147362

[93] Odén A McCloskey EV Kanis JA Harvey NC Johansson H. Burden of high fracture probability worldwide: secular increases 2010-2040. Osteoporosis. (2015) 26:2243–8. 10.1007/s00198-015-3154-6 26018089

[94] Ismail AA Cockerill W Cooper C Finn JD Abendroth K Parisi G. Prevalent vertebral deformity predicts incident hip though not distal forearm fracture: results from the European Prospective Osteoporosis Study. Osteoporosis. (2001) 12:85–90. 10.1007/s001980170138 11303719

[95] Giangregorio LM Papaioannou A Macintyre NJ Ashe MC Heinonen A Shipp K. Too Fit To Fracture: exercise recommendations for individuals with osteoporosis or osteoporotic vertebral fracture. Osteoporosis. (2014) 25:821–35. 10.1007/s00198-013-2523-2 24281053 PMC5112023

[96] Wilson N Hurkmans E Adams J Bakkers M BaláŽová P Baxter M. Prevention and management of osteoporotic fractures by non-physician health professionals: a systematic literature review to inform EULAR points to consider. RMD open. (2020) 6:1143. 10.1136/rmdopen-2019-001143 32144136 PMC7059534

[97] Nyman SR Ballinger C Phillips JE Newton R. Characteristics of outdoor falls among older people: a qualitative study. BMC Geriatr. (2013) 13:125. 10.1186/1471-2318-13-125 24245830 PMC3835551

[98] Spiegl U Bork H Grüninger S Maus U Osterhoff G Scheyerer MJ. Osteoporotic fractures of the thoracic and lumbar vertebrae: diagnosis and conservative treatment. Dtsch Arztebl Int. (2021) 118:670–7. 10.3238/arztebl.m2021.0295 34342263 PMC8727857

[99] Gibbs JC MacIntyre NJ Ponzano M Templeton JA Thabane L Papaioannou A. Exercise for improving outcomes after osteoporotic vertebral fracture. Cochrane Database Syst Rev. (2019) 7:Cd008618. 10.1002/14651858.CD008618.pub3 31273764 PMC6609547

[100] Sanghani-Kerai A McCreary D Lancashire H Osagie L Coathup M Blunn G. Stem cell interventions for bone healing: fractures and osteoporosis. Curr Stem Cell Res Ther. (2018) 13:369–77. 10.2174/1574888X13666180410160511 29637866

[101] Jiang Y Zhang P Zhang X Lv L Zhou Y. Advances in mesenchymal stem cell transplantation for the treatment of osteoporosis. Cell Prolif. (2021) 54:e12956. 10.1111/cpr.12956 33210341 PMC7791182

[102] Su P Tian Y Yang C Ma X Wang X Pei J. Mesenchymal stem cell migration during bone formation and bone diseases therapy. Int J Mol Sci. (2018) 19:2343. 10.3390/ijms19082343 30096908 PMC6121650

