# Supplementary material for: Global trends and hotspots in research on osteoporosis rehabilitation: A bibliometric study and visualization analysis
Source: Front Public Health. 2022 Nov 30;10:1022035. doi: 10.3389/fpubh.2022.1022035 (PMC9748484; doi:10.3389/fpubh.2022.1022035)
Supplement: Supplementary file 1 [file Table_1.DOC]

Supplementary 1 Related search terms in this article

| Topic | Search terms |
| --- | --- |
| Rehabilitation therapy -related topic | ＂physical medicine＂OR＂physical activity＂OR＂Self Care＂OR＂Self-Management＂OR＂Dance Therapy＂OR　Rehabilitation　OR＂Recreation Therapy＂OR　Telerehabilitation　OR＂Physical Therapy＂OR＂Activities of Daily Living＂OR＂Early Ambulation＂OR　Exercises　OR＂Physical Exercise＂OR＂Exercise Therapy＂OR＂Aerobic Exercise＂OR＂High-Intensity Interval Training＂OR＂High-Intensity Intermittent Exercise＂OR＂Resistance Training＂OR＂strength training＂OR＂balance exercise＂OR＂weight-bearing exercises＂OR＂Endurance Training＂OR＂Muscle Stretching Exercises＂OR＂Rehabilitation Exercises＂、Running、Swimming、Walking　OR　Pilates　OR　yoga　OR＂Wuqinxi exercise＂OR＂Physical Therapy Modalities＂OR＂Electric Stimulation Therapy＂OR＂Therapeutic Electrical Stimulation＂OR＂Electrical Stimulation Therapy＂OR＂Rehabilitation Exercise＂OR＂Remedial Exercises＂OR　Electrotherapy　OR＂Extracorporeal Shockwave Therapy＂OR＂Extracorporeal Shockwave Therapies＂OR＂Shock Wave Therapy＂OR＂Shock Wave Therapies＂OR＂Extracorporeal Shock Wave Therapy＂OR　ESWT OR PEMF　OR＂pulsed electromagnetic fields＂OR＂Low-Intensity Pulsed Ultrasound＂OR＂whole body vibration＂ |
| Osteoporosis-related topic | Osteoporoses　OR　Osteoporosis　OR＂Bone Loss＂OR＂Bone Losses＂OR＂Senile Osteoporoses＂OR＂Senile Osteoporosis＂OR＂Age-Related Osteoporosis＂OR＂Age-Related Bone Loss＂OR＂Age-Related Bone Losses＂OR＂Perimenopausal Bone Loss＂OR＂Postmenopausal Bone Losses＂OR＂Post-Menopausal Osteoporosis＂OR＂Postmenopausal Osteoporosis＂OR＂Perimenopausal Bone Losses＂OR＂Postmenopausal Bone Loss＂OR＂Post-Traumatic Osteoporoses＂OR＂Post-Traumatic Osteoporosis＂ |
