# Supplementary material for: Global trends and hotspots in research on osteoporosis rehabilitation: A bibliometric study and visualization analysis
Source: Front Public Health. 2022 Nov 30;10:1022035. doi: 10.3389/fpubh.2022.1022035 (PMC9748484; doi:10.3389/fpubh.2022.1022035)
Supplement: Supplementary file 2 [file Table_2.DOC]

Supplementary 2 The number of publications involved in the osteoporosis rehabilitation field from 1965 to 2022

| NO | year | publications | percentile of 3268 | NO | year | publications | percentile of 3268 |
| --- | --- | --- | --- | --- | --- | --- | --- |
| 1 | 2022 | 81 | 2.479 | 24 | 1999 | 55 | 1.684 |
| 2 | 2021 | 197 | 6.03 | 25 | 1998 | 78 | 2.388 |
| 3 | 2020 | 180 | 5.51 | 26 | 1997 | 58 | 1.775 |
| 4 | 2019 | 156 | 4.775 | 27 | 1996 | 55 | 1.684 |
| 5 | 2018 | 168 | 5.142 | 28 | 1995 | 66 | 2.02 |
| 6 | 2017 | 142 | 4.346 | 29 | 1994 | 66 | 2.02 |
| 7 | 2016 | 146 | 4.469 | 30 | 1993 | 67 | 2.051 |
| 8 | 2015 | 158 | 4.836 | 31 | 1992 | 42 | 1.286 |
| 9 | 2014 | 158 | 4.836 | 32 | 1991 | 35 | 1.071 |
| 10 | 2013 | 133 | 4.071 | 33 | 1990 | 5 | 0.153 |
| 11 | 2012 | 126 | 3.857 | 34 | 1989 | 7 | 0.214 |
| 12 | 2011 | 112 | 3.428 | 35 | 1988 | 2 | 0.061 |
| 13 | 2010 | 123 | 3.765 | 36 | 1987 | 3 | 0.092 |
| 14 | 2009 | 100 | 3.061 | 37 | 1986 | 1 | 0.031 |
| 15 | 2008 | 109 | 3.336 | 38 | 1985 | 1 | 0.031 |
| 16 | 2007 | 97 | 2.969 | 39 | 1984 | 5 | 0.153 |
| 17 | 2006 | 102 | 3.122 | 40 | 1983 | 3 | 0.092 |
| 18 | 2005 | 71 | 2.173 | 41 | 1982 | 2 | 0.061 |
| 19 | 2004 | 75 | 2.296 | 42 | 1979 | 1 | 0.031 |
| 20 | 2003 | 80 | 2.449 | 43 | 1978 | 1 | 0.031 |
| 21 | 2002 | 61 | 1.867 | 44 | 1976 | 1 | 0.031 |
| 22 | 2001 | 67 | 2.051 | 45 | 1974 | 1 | 0.031 |
| 23 | 2000 | 69 | 2.112 | 46 | 1965 | 1 | 0.031 |
|  |  |  |  |  |  |  |  |
