# Supplementary figures and images for: Global trends and hotspots in research on osteoporosis rehabilitation: A bibliometric study and visualization analysis
Source: Front Public Health. 2022 Nov 30;10:1022035. doi: 10.3389/fpubh.2022.1022035 (PMC9748484; doi:10.3389/fpubh.2022.1022035)

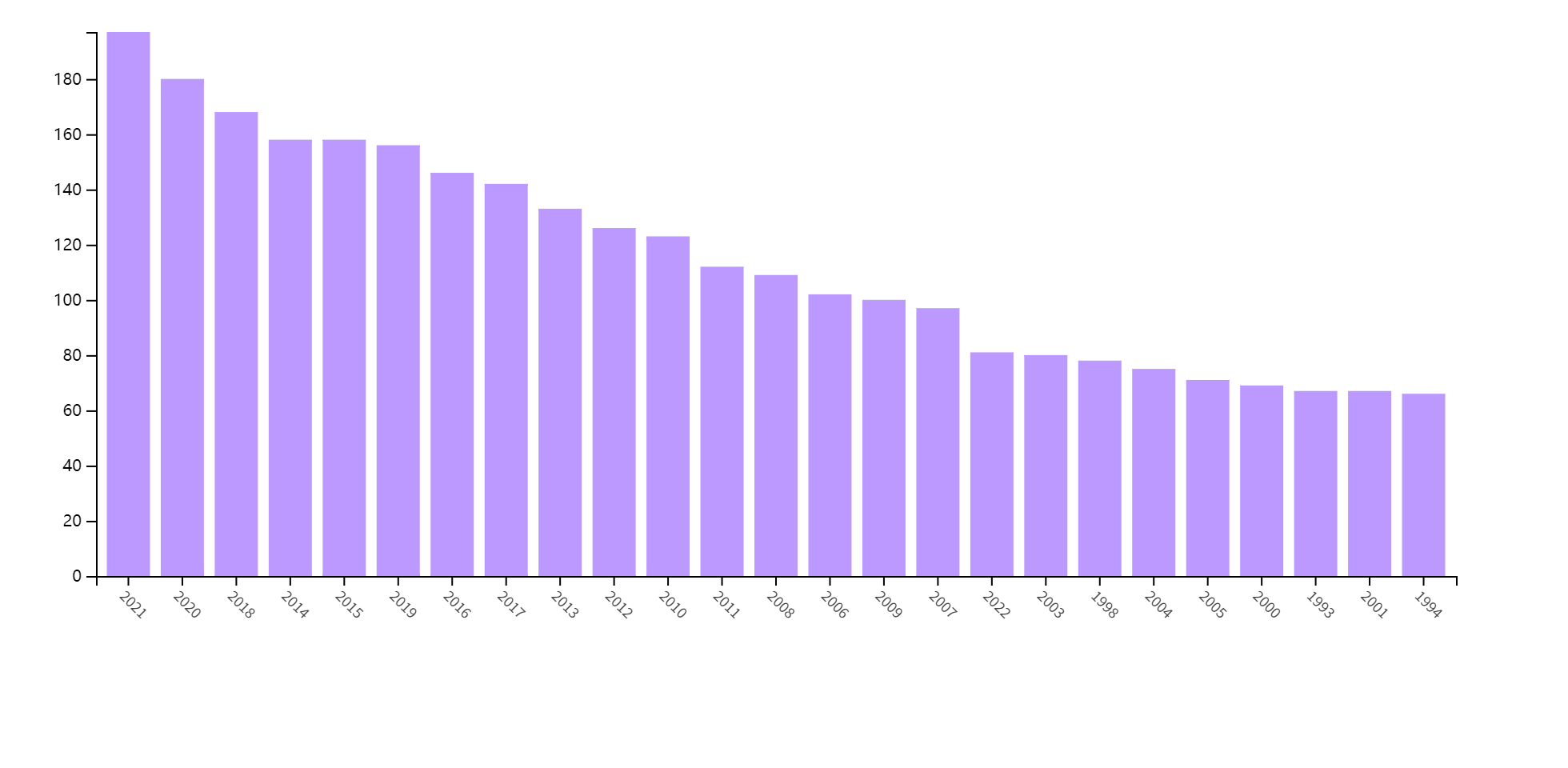

Supplement: Supplementary file 3 [file Image_1.JPEG]

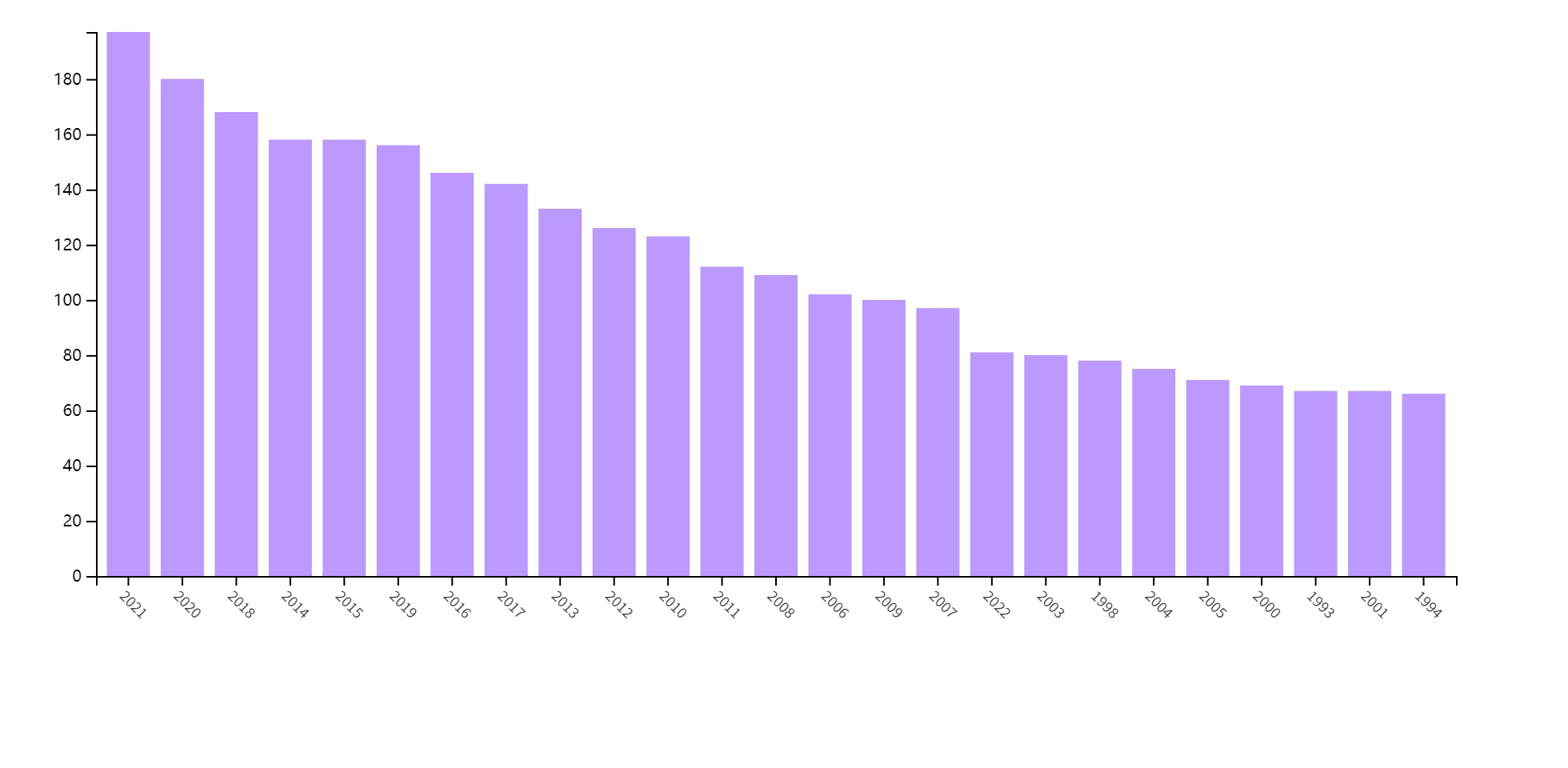

Supplement: Supplementary file 4 [file Image_2.PNG]
